# Extended Reality for Mental Health Evaluation: Scoping Review

**DOI:** 10.2196/38413

**Published:** 2024-07-24

**Authors:** Olatunji Mumini Omisore, Ifeanyi Odenigbo, Joseph Orji, Amelia Itzel Hernandez Beltran, Sandra Meier, Nilufar Baghaei, Rita Orji

**Affiliations:** 1 Research Centre for Medical Robotics and Minimally Invasive Surgical Devices Shenzhen Institutes of Advanced Technology Chinese Academy of Sciences Shenzhen China; 2 Faculty of Computer Science Dalhousie University Halifax, NS Canada; 3 Department of Psychiatry Dalhousie University Halifax, NS Canada; 4 School of Electrical Engineering and Computer Science University of Queensland St Lucia Australia

**Keywords:** extended reality, mental disorder, depression, anxiety, exposure therapy

## Abstract

**Background:**

Mental health disorders are the leading cause of health-related problems worldwide. It is projected that mental health disorders will be the leading cause of morbidity among adults as the incidence rates of anxiety and depression grow worldwide. Recently, “extended reality” (XR), a general term covering virtual reality (VR), augmented reality (AR), and mixed reality (MR), is paving the way for the delivery of mental health care.

**Objective:**

We aimed to investigate the adoption and implementation of XR technology used in interventions for mental disorders and to provide statistical analyses of the design, usage, and effectiveness of XR technology for mental health interventions with a worldwide demographic focus.

**Methods:**

In this paper, we conducted a scoping review of the development and application of XR in the area of mental disorders. We performed a database search to identify relevant studies indexed in Google Scholar, PubMed, and the ACM Digital Library. A search period between August 2016 and December 2023 was defined to select papers related to the usage of VR, AR, and MR in a mental health context. The database search was performed with predefined queries, and a total of 831 papers were identified. Ten papers were identified through professional recommendation. Inclusion and exclusion criteria were designed and applied to ensure that only relevant studies were included in the literature review.

**Results:**

We identified a total of 85 studies from 27 countries worldwide that used different types of VR, AR, and MR techniques for managing 14 types of mental disorders. By performing data analysis, we found that most of the studies focused on high-income countries, such as the United States (n=14, 16.47%) and Germany (n=12, 14.12%). None of the studies were for African countries. The majority of papers reported that XR techniques lead to a significant reduction in symptoms of anxiety or depression. The majority of studies were published in 2021 (n=26, 30.59%). This could indicate that mental disorder intervention received higher attention when COVID-19 emerged. Most studies (n=65, 76.47%) focused on a population in the age range of 18-65 years, while few studies (n=2, 3.35%) focused on teenagers (ie, subjects in the age range of 10-19 years). In addition, more studies were conducted experimentally (n=67, 78.82%) rather than by using analytical and modeling approaches (n=8, 9.41%). This shows that there is a rapid development of XR technology for mental health care. Furthermore, these studies showed that XR technology can effectively be used for evaluating mental disorders in a similar or better way that conventional approaches.

**Conclusions:**

In this scoping review, we studied the adoption and implementation of XR technology for mental disorder care. Our review shows that XR treatment yields high patient satisfaction, and follow-up assessments show significant improvement with large effect sizes. Moreover, the studies adopted unique designs that were set up to record and analyze the symptoms reported by their participants. This review may aid future research and development of various XR mechanisms for differentiated mental disorder procedures.

## Introduction

### Background

Mental disorders are defined as behavioral or mental patterns that cause significant distress or impairment for an individual. These are highly prevalent and, currently, are the leading cause of disability worldwide. In the past decades, a worldwide increase in the incidence of mental disorders has been observed [1,2]. According to the World Health Organization (WHO), mental disorders are the leading cause of disability in the United States and the United Kingdom. The WHO predicted that mental disorders would account for 13% of the total burden of diseases by 2030 [3]. As an indication, around 20% of adults experience 1 type of mental health problem in the United States, the United Kingdom, and related high-income countries [4]. A recent survey shows that the acceleration of socioeconomic developments has increased the prevalence of mental disorders (17.5% adults) in China [5]. Meanwhile, adolescents are characterized with the highest incidence of mental disorders in Canada [6]. Although over 75% of people with mental disorders remain untreated in middle-income countries, 35%-50% of the corresponding range is also found in high-income countries [3].

The WHO estimated that these mental conditions will cost the worldwide economy about US $1 trillion in lost productivity annually [7]. Some of the mental disorders are interlinked. For instance, anxiety and depression remain the most common mental disorders in society [8]. Anxiety is closely linked to mood disorders, and individuals developing depression have often experienced anxiety disorder at some earlier point(s) in life [9]. Although the etiology of anxiety disorder and depression is complex, multiple causal factors, such as rapid social change, stressful work conditions, gender discrimination, social exclusion, an unhealthy lifestyle, physical ill health, human rights violations, and genetics, have been appropriately studied. Many times, mental health researchers have studied the positive effects of evaluating anxiety in combination with other mental conditions, such as pain and depression. For example, Bandelow and Michaelis [10] reported that 1 of every 13 mental disorders is anxiety with major depressive and specific phobia disorder. In general, reports mostly suggest that closer mental care should be addressed by increasing the accessibility and development of tools that patients can use on their own [3].

### Conventional Assessment Approaches

Cognitive behavioral therapy (CBT) is a conventional approach that is shown to be effective in the treatment of a wide range of mental disorders, such as anxiety disorder, depression, phobia, and alcohol use problems [11]. CBT is based on the core principle that thoughts impact feelings and feelings impact behavior. During CBT, patients learn to change maladaptive thinking patterns and novel coping behaviors to become and stay healthy. CBT can be as effective as, or even more effective than, other forms of psychological therapy or even psychiatric medications, especially for patients diagnosed with anxiety disorder or depression. CBT is well supported by many clinical practice guidelines [11]. Studies have shown that it is an evidence-based therapy that reliably helps in overcoming depression. However, it involves aiding people to identify and change the bad lifestyles that negatively influence their behavior and emotions [12]. Rather than being a set method, CBT combines procedures that are developed on a certain disorder that has been unevaluated. For instance, the treatment procedure for depression is different from how CBT is used in evaluating phobia and anxiety disorder.

Exposure therapy is a major element of CBT that is more focused on certain mental disorders related to anxiety [13]. In this approach, participants, the subjects being assessed, are exposed to feared objects, activities, or situations in a safe environment, and this is known to reduce patients’ fear and possibility of avoidance. With gradual follow-up, participants learn to overcome their anxiety [14]. The variations of exposure therapy can be majorly classified as conventional exposure and modern exposure, usually based on the application context. Conventional exposure includes both in vivo and imaginal exposure. During in vivo exposure, patients are intentionally faced with real-world objects or situations they fear to reduce their anxiety [15]; however, it only works in a small percentage of mental health cases. In contrast, imaginal exposure configures an alternative approach during which patients imagine the worst outcome scenarios to confront their fears within their mind. The effectiveness of imaginal exposure depends on a patient’s motivation and their ability to generate fear-inducing imaginations. Exposure therapy is challenging as therapists require extensive training and multiple, long exposure sessions. Consequently, the conventional methods are time-consuming and costly. Recently, XR was evaluated as a new approach for delivering exposure-based therapy for mental disorders. The potential of XR for treating anxiety and depression has been reported [16,17].

### Use of Extended Reality in Mental Health

“Extended reality” (XR) is an umbrella term referring to all real, virtual, and mixed environments, wherein interactions are generated by computer technology to engage humans [13]. XR is a rapidly growing technology and is playing prominent roles in different sectors, such as providing clear benefits in many aspects of work and business, including training, collaborative working, and marketing. The technology is rapidly gaining traction in creating imagination of real worlds through virtual reality (VR), augmented reality (AR), and mixed reality (MR). XR was recently conceived for carrying out the evaluation of mental health. Thus, patients with mental disorders can be virtually immersed to allow them to display and confront the disorders they have. It has been noted that the advances in XR tools can transform the health domain remarkably; however, an exciting issue is studying the adoption and implementation levels of current VR, AR, and MR techniques for evaluating mental health [18-20]. In the industry sector, reports showed that the XR medical market was estimated to reach US $1.7 billion in 2022, with a compound annual growth rate of 105.6% from 2018 to 2022 [21]. Thus, supporting XR-based solutions will play a crucial role in the future of mental health. As the market continues to grow, it is safe to assume that developing XR technologies for mental disorder interventions will continue to increase.

In mental health interventions, XR techniques involve the use of single or multiple base technologies to create exposure. The base technologies, namely VR, AR, and MR, involve using computer models to artificially design real-world environments with stimuli sensory features. Thus, the artificial environment can simulate typical contexts that induce mental disorders, such as anxiety, phobia, or pain, to enable users to interact with the environment. Typically, an artificial environment can be developed using 4 main components:

- A high-end graphics-rendering unit that is used to compute and render virtual scenes via a frame buffer
- A 3D stereo display unit that connects users’ visual sensory system to the environment
- A tracking system that models users’ movement in the virtual environment
- Other input interfaces, such as joysticks or sensory gloves, that provide tactile feedback

Currently, studies suggest that XR-based evaluations can be as effective as conventional exposure–based methods [11,22-24]. It is anticipated that XR technology will offer the greatest promise for mental health care [11]. This is because XR-based exposure therapies are found to be accessible and can offer lasting improvements for different mental health conditions. By analyzing many studies, we have found that a good number of XR techniques exist. These are used to evaluate different mental disorders via different software and hardware technologies [11,22,25,26]. XR systems have been successfully applied in individual, group-based, and internet-based mental health interventions [27-29]. The adoption of XR systems started around 2 decades ago, when Hoffman and coworkers [30] developed a VR gaming system called *SnowWorld* for exposure-based therapy in mental health care. The game provides a systematic way of reducing players’ pain perception during burn wound care. Anderson et al [22] presented a follow-up of the first randomized clinical trial to test another format for delivering CBT for social anxiety disorder—VR exposure therapy. The study showed that VR and exposure group therapy has been well established as an effective strategy for evaluating social anxiety disorder.

The application of XR technologies (VR, AR, MR) for mental health care delivery provides opportunities and a greater degree of control for therapists to customize, reproduce, and tweak several evaluation parameters according to an individual patient’s needs during mental health care. Such parameters include fan wind, stereo sound, a moving chair, a color display, and odor emitters [31]. This kind of customization may not be achieved in traditional exposure therapy [32,33]. In addition, the risks associated with privacy intrusion reduce as everything is transformed into a virtual environment [34]. Simulated and augmented environments are less scary than the use of in vivo and imaginal exposure in conventional therapy [30]. Exposure-based therapies defined on VR/AR/MR apps have been shown to be effective for evaluating different mental health conditions. This study presents the findings of a scoping review of the state-of-the-art XR systems used in mental health care.

### Objective

XR-based mental health interventions have been advancing rapidly. It is critical to analyze the implementation and adoption levels of state-of-the-art XR techniques (ie, a combination of studies that have reported VR, AR, and MR) used for mental health care delivery. This review was conducted following the guidelines outlined by Arksey and O’Malley [35]. The main objective of this scoping review was to show the implementation and usage levels of XR-based therapy in providing care for different mental disorders worldwide. Thus, this review was set to provide a statistical analysis of studies that have recently focused on (1) technological design and usage of XR in mental health care with a worldwide demographic focus, (2) components that are found in different XR interventions used for mental disorders, and (3) effectiveness of XR technology in anxiety and depression as top mental disorders.

## Methods

### Eligibility Criteria

The adoption of VR, AR, and MR for mental disorder evaluation has evolved over time. The rapid advancement has occurred in a corresponding timeline with developments in the hardware and software used for implementing XR technologies. Hence, we decided to limit our data sources to papers published between August 2016 and December 2023 so as to analyze the state of the art in the study area. Advance search sections of 3 databases by the authors OM, IO, JO, and AB individually and the papers located were later combined to ensure a wide coverage of papers published in the search period. Further, only a limited set of search criteria were used to limit the papers extracted to more relevant ones. We only considered studies that were published in peer-reviewed journals and refereed conferences (with oral presentations). Ten records were identified via professional sourcing. Based on our search strategy and study goal, we decided to use a combination of 2 search rules: (1) all search terms must be present in the paper’s title or abstract or both, and (2) the paper’s publication year must be within the specified range between August 2016 and December 2023. Additionally, exclusion criteria were defined as (1) duplicate papers; (2) version updates; (3) papers written in a language other than English; (4) studies that reported anxiety or depression as a secondary aspect or induced illness; and (5) papers presenting strengths, weaknesses, opportunities, threats (SWOT) analysis, thesis and citations, and scoping reviews.

### Information Sources

We formulated a search strategy used to explore multiple databases to find all recent and relevant studies on XR technologies. We focused on information from studies that focused on depression and anxiety and related mental disorders. The scoping search was conducted on 3 different databases that are library sources for research papers, gray literature, patents, and common information. Our choices of databases were (1) PubMed, (2) Google Scholar, and (3) the ACM Digital Library. These databases were chosen because they provide an interface to generate wild search queries across a variety of disciplines, databases, and journals. In addition, they have the most complete indexes of papers that focus on the theme of this review study. This aided us in simultaneously accessing a broad range of evidence, including technical and peer-reviewed studies reported from different parts of the world, different publishers, and over a long period. We defined our search period to filter out only papers published between August 2016 and December 2023 and indexed in any of the 3 databases. Overlapping papers were filtered to avoid duplication. Multilevel filtering was carried out following PRISMA (Preferred Reporting Items for Systematic Reviews and Meta-Analyses) guidelines [35] and the associated checklist in Multimedia Appendix 1. This limited the search outcomes to relevant studies that could provide the most valuable data to answer our research objective. The search strategy was set to limit data sources to studies that implemented or used VR, AR, or MR for different mental disorders.

### Search Strategy

Our paper search strategy was based on using an organized structure of search terms to retrieve the existing literature in the 3 databases. We combined the keywords in our research objective in order to retrieve relevant papers from the databases. The search terms were discussed among the research team members and were defined as “augmented reality,” “mixed reality,” “virtual reality,” “depression,” “anxiety,” and “mental health.” We chose the free-text search as it is more flexible, and targeting the free words in both the title and abstract fields to limit the final sets provided an efficient way to increase the specificity of our search. These keyword searches were carried out for each concept in our research objective, and we designed the search queries to include a combination of the Boolean operators “OR” and “AND” to reduce the omission of vital papers. These search terms were the most appropriate keywords that were reflected in the subject area and had the utmost relevance to our review objective. We exclusively used full terms during the search in order to avoid any potential conflicts with other terms; for example, VR for “virtual reality” might also be used for “voice recognition,” which would make the filtering cumbersome and not necessarily generate additional useful resources. The selection criteria were carefully designed to consider papers that contained 1 or multiple search terms in the their title or abstract or both.

### Study Selection

**Figure 1:**
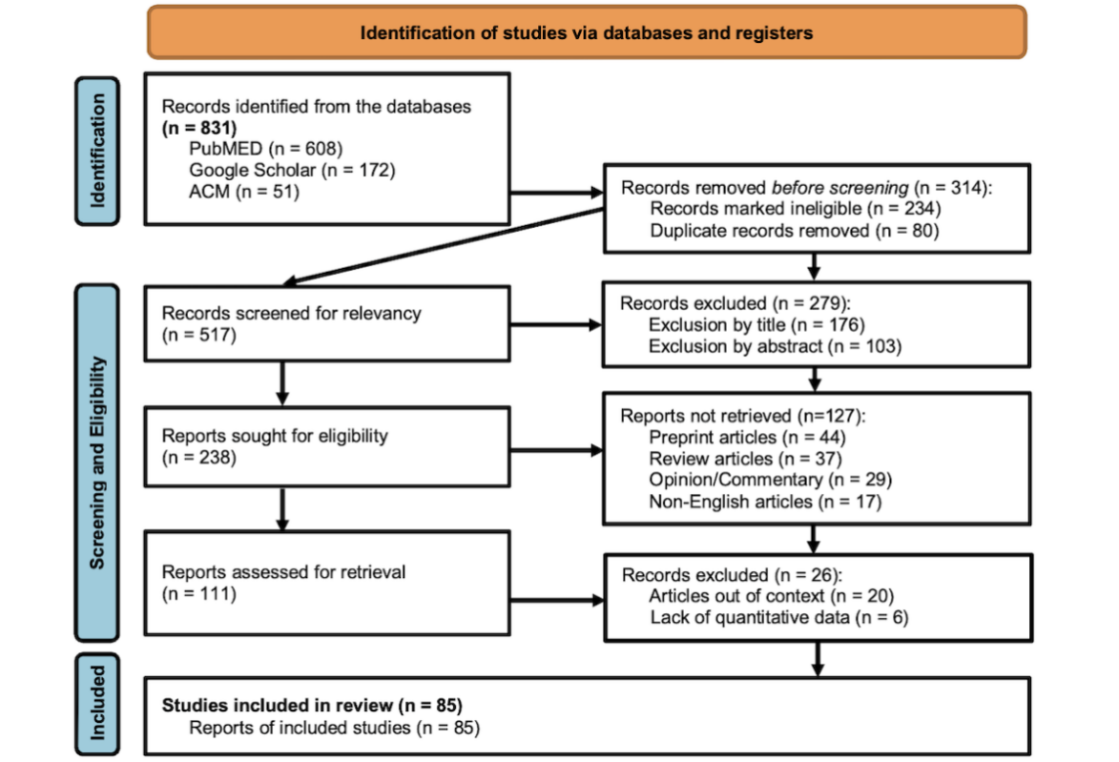
Literature screening and selection flowchart following PRISMA guidelines. PRISMA: Preferred Reporting Items for Systematic Reviews and Meta-Analyses.

The search yielded 831 papers scraped from the 3 databases, and 10 papers were identified through a professional source. Specifically, by using the defined search filter criteria “anywhere in the article,” the search results included 608 (73.16%), 172 (20.7%), and 51 (6.14%) papers scraped from PubMed, Google Scholar, and the ACM Digital Library, respectively. The papers were scraped and processed by following the set of items in the PRISMA checklist. First, authors IO, JO, and AB independently screened the papers retrieved, while author OM performed a quality check on all the final records. Next, irrelevant and duplicate papers (314/831, 37.79%) were removed; thus, a total number of 517 (61.47%) papers were left. The remaining papers were further screened for relevance. With title screening, 176 (30.04%) irrelevant papers were removed. Full abstract reviews was performed in situations in which a paper’s relevance could not be resolved from its title. Thus, 103 (58.52%) papers were further screened out. Yet, authors carried out a review of the full text when certainty on a paper’s relevance was still lacking in order to decide whether it was relevant. In total, 279 (53.97%) nonrelevant papers were screened out by the authors, leaving only 238 (46.03%) papers for retrieval. A second screening step was required to limit the scoping review to papers that fulfilled the eligibility criteria. Thus, further assessment was carried out, and another 127 (53.36%) papers were removed. The full texts of the remaining 111 (46.64%) papers were retrieved. Papers that were out of context (n=20, 18.02%) and those that lacked quantitative data (n=6, 5.4%) were also excluded. Finally, a total of 85 (76.58%) papers that meet the eligibility criteria were included in this review. All these procedures were performed in Microsoft Excel and without any form of automation. The paper selection process strictly followed the steps shown in Figure 1.

### Data Collection and Information Extraction

Three of the authors performed data extraction, while data validity and accuracy were checked by a fourth author. The full texts of the 85 papers were downloaded and shared among the authors for review. The following specific details of the papers were extracted and processed in Excel to analyze the adoption of VR, AR, and MR in managing anxiety and depression and related mental disorders:

- Authors, year, and regions
- The study type and study design focus and health domain
- the methodology (eg, study duration, number of sessions, and duration in minutes)
- The methodology that the study was based on and the evaluation strategy
- The VR/AR/MR app and technology (eg, type of headset, toolkit) used for the study
- Study demographics, such as targeted population, sample size, and age distribution
- Motivational strategies, targeted outcome, and region
- Key findings on using the XR techniques for managing depression and anxiety

The details related to the abovementioned data were used to address the specific research objective guiding this scoping review. The useful insights provided by the data could help developers and researchers on future research on VR/AR/MR for the intervention of mental disorders. In addition, users can learn the importance of such systems, such as the use of XR-based exposure therapy, for mental health.

## Results

### Publications’ Demographics by Country and Year

This scoping review was based on a total of 85 papers [26,32,36-118], which are described in Multimedia Appendix 2*.* We reported the statistics and meta-analysis of studies that addressed the technological design and usage of XR in mental health, the major components used in different XR interventions for the management of mental disorders, and the effectiveness of the XR technology in anxiety and depression as top mental disorders.

First, we analyzed the country of origin of the papers. The 85 studies were conducted in 27 countries worldwide, as presented in Table 1. Of these 85 studies, 14 (16.47%) were carried out in the United States, followed by 11 (12.94%) in Germany. Compared to a previous study [12], both countries dedicated a good amount of research funding and time to study how XR aids mental health care in the United States and Germany. The data also showed that a good number of studies were conducted in South Korea (n=8, 9.41%) and the Netherlands (n=6, 7.06%). Our study infers that compared to the remaining 21 countries, the aforementioned 4 countries invested a good amount of effort in domestic technological development toward creating XR-based tools for mental disorder. Thus, XR systems contribute immensely to the economic and health care systems of high-income countries. Meanwhile, our data also identified that mental studies are not yet prioritized in Africa. In terms of study frequency by year, Table 2 shows that the majority of papers (n=27, 31.76%) were published in 2021. This could underline a worldwide priority set to advance mental health care. However, none of the studies mentioned whether this was attributed to the ongoing COVID-19 pandemic [119]. Nonetheless, secondary studies identified that the prevalence of anxiety and depression increased by 25% in the first year of the pandemic, while the psychosocial effects of the pandemic varied by regions [120,121]. Hence, it is likely that the pandemic-related increase in mental disorders and the increased adoption of virtual treatment during the pandemic contributed to the rise in the number of XR-based mental health interventions in 2021. Furthermore, there was a great decline in the number of investigations reported in 2022 (n=12, 14.12%) and 2023 (n=16, 18.82%). This coincides with the time COVID-19’s worldwide prevalence had decreased.

**Table 1:** List of countries that conducted mental health studies using XR^a^ technologies.

Number	Country of study	Studies (N=85), n (%)
1	Armenia	1 (1.18)
2	Australia	2 (2.35)
3	Austria	2 (2.35)
4	Belgium	1 (1.18)
5	Canada	1 (1.18)
6	China	4 (4.71)
7	Denmark	2 (2.35)
8	France	1 (1.18)
9	Germany	11 (12.94)
10	Hong Kong	1 (1.18)
11	India	1 (1.18)
12	Iran	3 (3.53)
13	Israel	2 (2.35)
14	Japan	1 (1.18)
15	Jordan	2 (2.35)
16	South Korea	8 (9.41)
17	Netherland	6 (7.06)
18	Philippines	1 (1.18)
19	Poland	2 (2.35)
20	Portugal	2 (2.35)
21	Romania	2 (2.35)
22	Singapore	1 (1.18)
23	Spain	7 (8.24)
24	Sweden	2 (2.35)
25	Turkey	1 (1.18)
26	United Kingdom	4 (4.71)
27	United States	14 (16.47)

^a^XR: extended reality.

**Table 2:** Number of studies published per year.

Year of study	Studies (N=85), n (%)
2016	3 (3.53)
2017	8 (9.41)
2018	4 (4.71)
2019	8 (9.41)
2020	7 (8.24)
2021	27 (31.76)
2022	12 (14.12)
2023	16 (18.82)

### Demographics of XR Usage: Age Population

We analyzed the age of the participants included in the studies and categorized the participants as *children*, *teenagers*, *adolescents*, *young adults*, *young and old adults*, and *old adults* based on the age ranges reported in the 85 studies included in this review. Studies that omitted such information were declared as *not specified*. A substantial age overlap was found among the groups of individuals included in the reviewed studies. Thus, *adults* were taken as participants between 18 and 65 years old in the reported studies, and *older adults* were above 65 years old. We observed that around half of the studies (n=38, 44.71%) were designed for adults (between 18 and 65 years old). However, a small number of studies focused on younger age groups: 10 (11.76%) of the 85 studies focused on participants between 0 and 12 years old, while 2 (2.35%) focused on participants in their teenage years as well. The poor representation of participants from each age group in the reviewed studies could be due to a lack of a standardized way of selecting a target audience when developing XR systems for managing mental disorders [122].

In Table 3, we indicated the common classification of participants’ age ranges reported in the selected studies, in addition to the statistical information derived from the age groups. This is because the age ranges used for defining the categories of participants in the 85 papers were not unique. In addition, there was great overlap when comparing the categories and age ranges across studies. Thus, we refined the data to synthesize the mean age distribution of the participants included in each study. For this, the age ranges were set as given or generated as (mean – SD) to (mean + SD), when only the mean (SD) was given. The mean age distributions of the participants used for classification are reported in Table 3. The data indicated that the majority of studies (n=47, 55.29%) were designed for an audience with a mean age of 35.079 (SD 9.72) years. The age distribution in this group was particularly dominated with lower and upper values of 18 years (26/47, 55.32%) and 65 years (7/47, 14.89%), respectively, in the different participants’ age ranges. The age range of the youngest participants who participated in an XR-based study on mental disorder [58] that investigated how VR reduces the perception of anxiety in infants were a group of children 4-8 years old.

**Table 3:** Table3. Participant categories found in the included studies by level of maturity.

Participant categories		Studies (N=85), n (%)
**Audience group**		
	Children	10 (11.76)
	Teenagers	2 (2.35)
	Adolescents	27 (31.76)
	Adults	38 (44.71)
	Older adults	3 (3.53)
	Not specified	5 (5.88)
**Age range (years)**		
	1-10	23 (27.06)
	11-20	47 (55.29)
	21-60	7 (8.24)
	61-85	1 (1.18)
	≥85	2 (2.35)
	Not specified	5 (5.88)
**Participants enrolled**		
	1-10	10 (11.76)
	11-20	9 (10.59)
	21-50	23 (27.06)
	51-100	22 (25.88)
	>100	16 (18.82)
	Not specified	5 (5.88)

### Demographics of XR Usage: Study Sample Size

We analyzed the sample size of participants enrolled in the 85 studies included in this scoping review. We divided the sample size into 5 different categories and analyzed the number of studies, as reported in Table 3. It can be seen that most studies recruited 51-100 participants (n=22, 25.88%). Next came studies that recruited 21-50 participants (n=23, 27.06%) to evaluate mental health care with XR. Furthermore, 16 (18.82%) studies included over 100 participants, while a small sample size (≤20 participants) was considered in 19 (22.35%) of the 85 studies. Most of these 19 studies were more subtle in their findings and conclusions. Thus, it can be understood that having relatively more participants is helpful to reach better conclusions. Overall, each of the participant categories identified was reported in at least 5 (5.88%) different studies. It is worth mentioning that only 5 (5.88%) studies did not specify the sample size used. The participants’ gender distributions were not analyzed, as these data were missing in most studies included in this scoping review.

### Demographics of Design and Implementation Strategies

The application of XR systems for mental disorders requires vigorous study and implementation strategies. We analyzed different factors usually considered when designing or evaluating XR systems for mental health interventions. The 3 major considerations found in the 85 selected studies were the *type* of study performed, *design* factors, and the *evaluation method* used to assess each study. With a focus on anxiety and depression, the 4 main types of studies that were are carried out were (1) *discussions*, which are studies with a narrative focus; (2) *experimental*, which are studies that are conducted to investigate the effect of XR techniques on certain groups of subjects or other factors that aid or affect such a setup; (3) *modeling*, which are studies that are conducted to develop new models or setups and validate these on limited subjects or data; and, lastly, (4) *analysis*, which are studies performed without any particular experimental study but relying on the data of previous *experimental* studies.

As reported in Table 4, it was found that 67 (78.82%) of the 85 studies investigated experimentally to report how XR aids in interventions for mental disorders. Meanwhile, 8 (9.41%) studies were based on modeling and analysis each, while 1 (1.18%) study was based on narration (ie, discussion). This shows that most studies were conducted as experimental investigations. Typically, this enabled a direct comparison between mental conditions and their relationships with their causal factors in psychological cornerstone studies [123]. Furthermore, 50 (58.82%) studies investigated the effects of XR immersion. This shows that researchers in this domain are commonly fond of investigating how immersion can influence mental health care procedures. The other design factors of the XR systems found in the 85 studies were on the subject’s process automation (n=14, 16.47%); these studies were majorly investigated to observe whether they well emulated real-world situations and environments. Similarly, 9 (10.59%) and 6 (7.06%) studies typically focused on cases of XR personalization and manual execution, respectively.

**Table 4:** Demographics of the study implementation factors.

Participant categories		Studies (N=85), n (%)
**Type of study**		
	Discussion	1 (1.18)
	Experimental	67 (78.82)
	Modeling	8 (9.41)
	Analysis	8 (9.41)
**Design factor**		
	Personalization	9 (10.59)
	Manual	6 (7.06)
	Process automation	14 (16.47)
	Immersion analysis	50 (58.82)
**Evaluation** **method**		
	Quantitative method	32 (37.65)
	Qualitative method	26 (30.59)
	Mixed method	23 (27.06)
	Not specified	4 (4.71)

### Relationship Between Study Periods and Duration per Session

**Figure 2:**
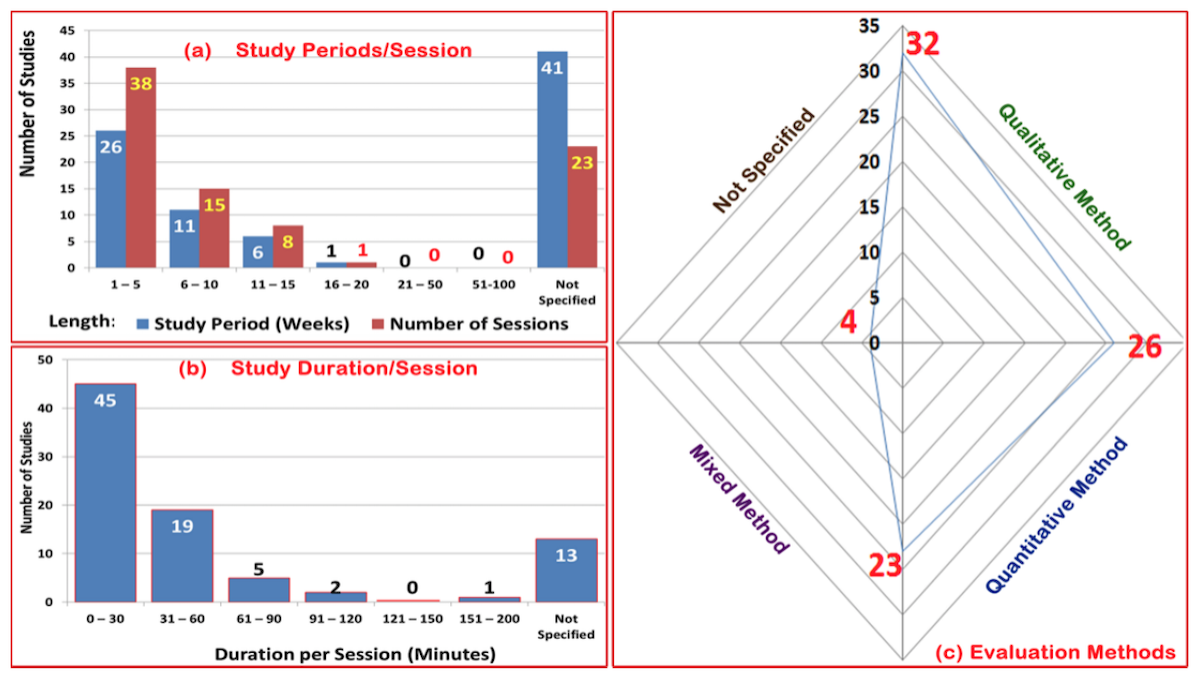
Categories of studies included in this review.

Next, we analyzed the common evaluation categories reported in the 85 studies. First, categories of study periods (in weeks) and the duration per session (in minutes) were analyzed with respect to the number of sessions in each study. As presented in Figure 2a, some of the studies (n=27, 31.76%) were carried out in 1-5 weeks, 7 (8.24%) studies lasted for 6-10 weeks, and 8 (9.41%) studies lasted for 11-15 weeks . It is worth emphasizing that the longest study (n=1, 1.18%) lasted for 16-20 weeks. In addition, 39 (45.88%) studies evaluated their participants in 1-5 sessions, while 15 (17.65%) studies evaluated their participants in 6-10 sessions.

Furthermore, we analyzed the duration per session (in minutes) for sessions that were reported in each study. In more than half of the studies (n=44, 51.76%), participants used XR techniques for 0-30 minutes, followed by studies requiring 31-60 minutes (n=19, 22.35%) and 61-90 minutes (n=5, 5.88%) of user engagement per session. On the extreme end, the XR technique was used for a single session that lasted over 100 minutes in 4 (4.71%) studies. It was also observed that 13 (15.29%) studies did not specify session durations.

In addition, we analyzed the common evaluation method used in the 85 studies included in this review, and identified 3 main methods: quantitative, qualitative, and mixed. The qualitative assessment approach was applied in 31 (36.47%) of the 85 studies, and it was understood that the qualitative method reveals deeper insights into XR-based evaluation. Furthermore, the quantitative method was used in 9 (10.59%) studies, while the mixed methods approach was used in 18 (21.17%) studies. We also found that 2 (2.35%) studies did not report evaluation methods (see Figure 2c).

### Extended Reality and Gamification Strategies for Mental Disorders

It is important to analyze the XR techniques used in interventions for depression and anxiety. First, we analyzed the major strategies found in the 85 studies included in this review. The XR tools used in each study were identified to be either gamified or nongamified. Gamification strategies were adopted in 26 (30.59%) studies, and these strategies were used across 10 mental disorders in all 85 papers. The few exceptions where a gamification strategy was not applied included negative thoughts, panic disorder, and pain and anxiety. Conversely, nongamified strategies were adopted in interventions for the remaining mental conditions, accounting for 59 (69.41%) of the 85 studies. However, alcohol use disorder and attachment behavior were only addressed using gamified XR systems. It may be right to think that gamification strategies are yet to mature for such conditions or, possibly, that existing gamification strategies are not suitable when evaluating such mental disorders using XR techniques or, perhaps, there are ongoing studies to show their applicability.

### Extended Reality Development Tools

We also analyzed XR apps that were used in the 85 studies and found that specific systems, such as 3D Unity Pro, are commonly used by many authors in the development of XR systems. Only 68 (77.64%) of the 85 studies reported the name of the actual XR app they implemented or adopted. As reported in Table 5, the most frequently used tool for developing XR platforms was 3D Unity Pro (n=16, 23.53%). This is probably due to its powerful editor to create XR systems and its support for cross-platform development. Similarly, we observed that some studies (n=15, 22.06%) were carried out with custom VR systems. Such adaptive apps are either newly developed or adopted and evaluated for aiding mental health care. Meanwhile, Blender 3D and mobile virtual systems were used in 3 (4.41%) and 10 (14.71%) studies, respectively. Another 24 (28.24%) studies indicated using a development platform but did not specify it, while the remaining 17 (20%) studies did not mention the use of any development platform.

**Table 5:** Software VR^a^ tools commonly used for XR^b^ development.

XR app	Studies (N=85), n (%)
3D Unity Pro	16 (18.82)
Custom adaptive VR software	15 (17.64)
Blender 3D	3 (3.52)
Mobile virtual system	10 (11.76)
Others	24 (28.24)

^a^VR: virtual reality.

^b^XR: extended reality.

### Hardware Technologies Used for XR in Mental Disorders

To further fulfill the aim of this study, we extracted information about the XR technologies used to deliver mental health care in the 85 studies. To be as inclusive as possible, we only reported the hardware components that were listed for setting up the XR environments in the studies (Table 6). The use of headsets was consistent in 46 (54.11%) of the 85 studies; thus, headsets are the most commonly used component when setting up XR for mental disorder interventions. Typically, it was found that the Oculus head-mounted display (HMD) and VR headsets were common in such studies. In addition, smartphones are a common technology used in setting up the XR environment. It was found that 7 (8.24%) studies included smartphones of different types. These hardware components (ie, HMDs, smartphones, and VR glasses) were increasingly popular in studies where the gamification strategy was adopted. We further analyzed the most popular types of headsets and found that they were headphones, earbuds, and VR HMDs. These last are a more advanced technology and a basic component in most XR studies. As reported in Table 7, there were 9 different types of HMDs used in the 85 studies. HTC Vive and Samsung Gear VR were the most used HMDs in setting up XR systems: these were found in 12 (14.12%) and 11 (12.94%) studies, respectively. The next such HMD was 3D VR glasses, which were used in 7 (8.24%) studies. In addition, Oculus Go and Google VR Box were used in 3 (3.53%) studies each; 2 (2.35%) papers reported to have used Oculus Rift; and different types of VR simulators, such as Oculus CV1, a custom electroencephalography (EEG) cap with a VR HMD, and the Windows MR headset were also used in 1 (1.18%) study each.

**Table 6:** Types of hardware technology used in XR^a^ interventions for mental disorders.

XR technology used	Studies (N=85), n (%)
VR^b^ HMD^c^	46 (54.11)
3D VR glasses	5 (5.88)
Smartphone	7 (8.24)
Google VR Box	3 (3.53)
EEG^d^/EMG^e^ cap	3 (3.53)
Headphones	3 (3.53)
Biopac MP150	2 (2.35)
Earbuds	2 (2.35)
Location tracker	2 (2.35)
Directional microphone	1 (1.18)
Gamepad	1 (1.18)
Webcam	1 (1.18)
Unspecified	9 (10.59)

^a^XR: extended reality.

^b^VR: virtual reality.

^c^HMD: head-mounted display.

^d^EEG: electroencephalography.

^e^EMG: electromyography.

**Table 7:** Types of VR^a^ headsets.

HMDs^b^ used	Studies (N=85), n (%)
HTC Vive	12 (14.12)
Samsung Gear VR	11 (12.94)
3D VR glasses	7 (8.24)
Oculus Go	3 (3.53)
Google VR Box	3 (3.53)
Oculus Rift	2 (2.35)
Oculus CV1	1 (1.18)
EEG^c^ VR HMD	1 (1.18)
Windows MR^d^ headset	1 (1.18)
Unspecified	5 (5.88)

^a^VR: virtual reality.

^b^HMD: head-mounted display.

^c^EEG: electroencephalography.

^d^MR: mixed reality.

### Extended Reality for Anxiety and Depressive Disorders

**Figure 3:**
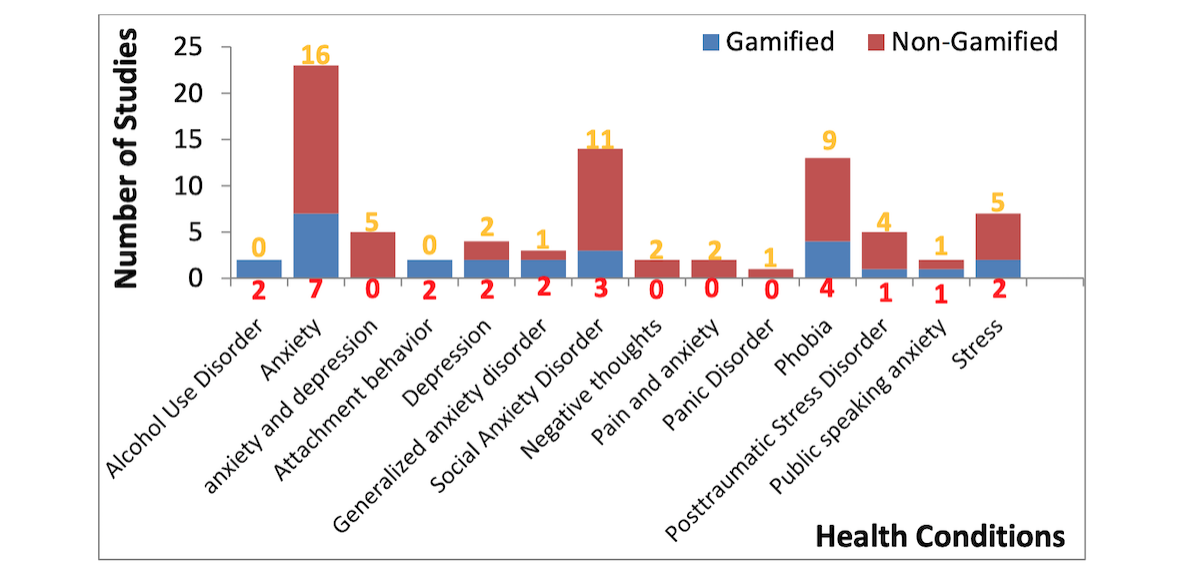
Number of studies per clinical condition.

We identified 14 types of mental disorders in the 85 papers and illustrated the number of studies investigating each condition, as shown in Figure 3. We observed that most of the XR studies were centered around anxiety and depression (n=53, 62.35%). These included the use of XR for anxiety without depression in 43 (52.94%) studies, where the primary and secondary focus included anxiety, and depression was not considered at all. In 4 (4.71%) studies, we found that minimal attention was on depression, wherein anxiety of any kind was not considered. Typically, 23 (27.05%) studies focused on just anxiety, while the remaining 30 (35.29%) studies combined anxiety with other mental disorders. Anxiety and depressive disorders sometimes have ambiguous borderline definitions; thus, this scoping review focused more on them. We further looked into individual conditions, such as social anxiety disorder and generalized anxiety disorder, which were found in 14 (16.47%) and 3 (3.53%) studies, respectively. These conditions were combined with unclassified distress in 2 (2.35%) studies [66,79]. Of the 85 papers included in this review, 13 (15.29%) were found to have applied XR technologies for phobia-related mental disorders (fear disorders that are a clinical evaluation of anxiety).

The adoption of XR for mental disorders without anxiety or depression was also studied. For instance, physiological disorders, such as emotion and stress issues, were investigated. Among these, phobias of different kinds (eg, acrophobia, claustrophobia, fear) were investigated in 13 (15.29%) of the 85 studies, and posttraumatic stress disorder was studied in 5 (5.88%) papers. The latter has a similar frequency as 1 of the top mental disorders, depression. We also found that negative thoughts and attachment behavior were investigated in 2 (2.35%) studies each. In contrast to Baghaei et al’s [124] findings, we found that generalized anxiety disorder was investigated as a specific clinical condition in 3 (3.53%) studies. In addition, other mental disorders, such as alcoholic use disorder, attention disorder, attachment behavior, negative thoughts, pain and anxiety, and public speaking anxiety, were found in only 2 (2.35%) studies, while panic disorder was found in only 1 (1.18%) study. Thus, XR technology is commonly used for evaluating anxiety disorder. Finally, this scoping study shows that XR-based evaluations are distinctly applied for anxiety and other mental disorders that exclude depression. A typical case includes the development of an XR system for anxiety and phobia, as well as anxiety and psychiatric disorders [16,87]. The benefits of XR for the evaluation and management of mental disorders were identified in the 85 papers that were reviewed. Recent studies show that VR yields the same level of effectiveness as exposure-based therapy for reducing anxiety symptoms [125]. This section mostly uncovered the use of VR technology in anxiety; however, it showed that AR and MR have been recently emphasized as an add-on technology and not a substitute. It is clear that more studies are still needed for evaluating how AR and MR can singly improve mental health.

## Discussion

### Principal Findings

In the previous section, we focused on the demographics, technologies, and study designs found in existing XR systems used in mental disorder interventions. In this section, the effectiveness of the XR systems for anxiety and depression as top mental disorders are analyzed, as reported in the 85 studies [26,32,36-118] included in this review.

#### Effectiveness of XR Technology for Mental Disorder Intervention

Following the review of the literature included in this scoping study, it can be concluded that XR systems are commonly used for managing mental disorders. In this scoping review, we found that XR technologies have been majorly used for evaluating anxiety and depression separately, in combination with each other or with other common mental disorders. In the latter case, the majority of studies were targeted at cognitive and behavioral change (ie, subjective care) to improve patients’ behavior or attitude or both. In addition, it was observed that among XR technologies, VR-based systems are mostly used. For instance, in some studies [26,77,82], VR was effectively used to evaluate anxiety and depressive symptoms in patients with mental disorders. Similarly, Li and Luo [84] established that gamified XR can reduce depressive disorders through cognitive empathy and mutual understanding among patients and caregivers. Many studies have reported that XR systems help reduce the symptoms of mental disorders. For instance, some authors [26,41,44] have strongly indicated that using XR technology in the psychotherapy process reduced anxiety and depression in their subjects. Similarly, Niharika et al [63] showed a significant decrease in subjects’ anxiety scores when using VR eyeglasses during dental treatment.

XR intervention is a safe, noninvasive technique that does not require any previous education and training and has lasting effects. However, McLay et al [59] showed that statistically significant differences between XR-based treatment and conventional approaches may not be a constant thing when applying XR systems for mental disorder intervention. In comparison to standard CBT, some authors [60,73] have improved the psychotherapy of depressive disorder in young adults by developing effective VR-enhanced personal construct therapy. Arnfred et al [101], in the SoREAL study, investigated in vivo group CBT and compared its effects with those of VR exposure CBT on patients diagnosed with social anxiety disorder. Similarly, Shin et al [107] and Donker et al [110] investigated the efficacy of mobile-based VR CBT for panic disorder and phobia interventions. The app-based XR interventions were effective in managing disorder symptoms and restoring subjects’ autonomic nervous system. This demonstrates the validity of using XR systems as self-guided and cost-effective therapeutic approaches. Taken together, these studies show that the recent development of XR technologies is gaining traction for mental disorder evaluation and treatment. Thus, some researchers have suggested that future XR interventions should consider providing multiuser experiences that can help increase social engagements for patients who are possibly confined due to disabilities. It can be concluded that virtual environments are as effective as exposure therapy for evaluating mental health. We found studies investigating whether gamified XR is also effective in reducing acrophobia, and the stimuli presented using AR, indeed, induced physiological alterations in the participants [43,44,80].

#### Effects of XR Design Factors on the Outcomes of Mental Disorder Interventions

Brás et al [78] showed that AR and VR offer high levels of immersion and are optimal solutions for counteracting the effects of in vivo exposure. Weerdmeester et al [74] showed that by engagement and cognitive biofeedback, gamified VR can reduce anxiety symptoms. Grieger et al [81] investigated whether XR-based interventions with multiple design factors can yield better results when used for mental disorders. In a randomized controlled trial, the authors found that personalized VR aids a general positive shift in thoughts and emotions, with increased relaxation and self-refection [81]. This shows that VR systems with multiple features, such as personalization, immersion and focus, interaction design and embodiment, and integration, can enhance treatment outcomes. Similarly, De Asis et al [82] developed a mobile VR-based system for promoting relaxation to reduce anxiety and alter stressful activities among class students. Studies conducted by El-Qirem et al [91] and Traister [79] have shown that XR technology can significantly lower students’ anxiety and enhance them psychologically and physiologically with a safe and risk-free therapeutic experience. The related studies [56,57] focused on mental stress management in teenagers and adolescents. Brivio et al [39] compared the efficiencies of 360° panorama technology and a computer-simulated prototype in generating an XR sense of presence, emotions, and relaxation when treating mental disorders. In addition, Lundin et al [98] investigated whether filming virtual environments with a low-cost 360° film camera to produce VR CBT can offer a feasible and acceptable treatment for some kinds of phobia. These studies show that VR exposure therapy can produce long-lasting benefits for mental disorders, consistent with research on a variety of forms of short-term CBT for social anxiety disorder. The results showed that treatment satisfaction was high and that participants had significant improvement at 6-month follow-up, with large effect sizes [98]. Another study [87] showed that VR relaxation induces positive affective states and has short-term effects toward reducing psychiatric stress and anxiety disorder symptoms compared to standard relaxation exercises.

### Limitations

XR-based systems have some unique advantages over traditional methods used for mental disorder management. Nevertheless, XR systems also have some limitations. Future developments should consider technological innovation and standardization of treatment options. The following limitations should be considered when interpreting the results of this review. The search strategy developed was limited to using PubMed, Google Scholar, and the ACM Digital Library databases for efficient and accurate search results. This may have excluded qualified papers from other databases. We also found that the number of weeks of evaluation in 40 studies and the number of sessions in 23 studies were not specified in the selected papers. Thus, it is difficult to assert the best number of weeks and sessions needed to validate the use of XR-based technology in mental disorder evaluation. This review identified various major methodological approaches and development tools used by studies. Another limitation of the study is the lack of scientific assessment of the quality of the publications that were included in the scoping review. Moreover, due to the large number of papers reviewed, there is a possibility of that we overlooked valid publications that might have met the inclusion criteria. Non-English papers were not included in this review either. Finally, considering the possibility of bias in the reported outcomes for many reasons, including due to self-reporting and publishing bias that tend to favor papers with positive outcomes, the findings of this scoping review should be applied with caution, especially regarding the effectiveness of XR-based intervention for mental disorders.

### Conclusion

XR therapy has been widely used in the care of a variety of mental disorders. This scoping review investigated the adoption of XR for mental disorders, specifically anxiety and depression. The review covered 85 studies that used different types of VR, AR, and MR technologies for mental disorders, with a focus on anxiety and depression. We uncovered that the majority of reviewed papers reported a reduction in the symptoms of anxiety or depression with the use of XR. Moreover, the studies adopted unique designs that were set up to monitor the signs of mental disorders. The recorded signs can be used for formulating appropriate therapies. We also found that XR-based interventions have been shown to be effective approaches with a high level of user acceptability in 18 mental health conditions. Although a considerable number of studies (N=85) were included in this scoping review, some areas are still underinvestigated and, hence, not well represented in the reviewed studies. For instance, the adoption of nongamified strategies was found to have cut across 18 mental health conditions included in this review. However, studies investigating pain and anxiety, negative thoughts, autoimmune disorders, and acquired brain injury did not use any form of gamification strategies. This study was conducted to investigate the implementation and adoption levels of XR for mental health care delivery. Our study outputs indicate that many studies have focused on anxiety, either alone or in combination with other conditions. Meanwhile, a limited number of studies have solely focused on depression. In a previous study, Baghaei et al [124] also showed that supporting people with depression in XR settings is an interesting area to explore for mental health care. We recommend that future work should conduct controlled trials to investigate and compare the effectiveness of using XR-based intervention in mental health care and the benefits and costs of XR in mental disorder management.

## Appendix

Multimedia Appendix 1 PRISMA (Preferred Reporting Items for Systematic Reviews and Meta-Analyses) checklist.

Multimedia Appendix 2 Table of studies.

## Abbreviations

AR: augmented reality
CBT: cognitive behavioral therapy
HMD: head-mounted display
MR: mixed reality
PRISMA: Preferred Reporting Items for Systematic Reviews and Meta-Analyses
VR: virtual reality
WHO: World Health Organization
XR: extended reality

## Data Availability

The data used for this review have been included as Multimedia Appendices with the manuscript.
