# Supplementary material for: Extended Reality for Mental Health Evaluation: Scoping Review
Source: JMIR Serious Games. 2024 Jul 24;12:e38413. doi: 10.2196/38413 (PMC11306946; doi:10.2196/38413)
Supplement: Multimedia Appendix 1 [file games_v12i1e38413_app1.docx]

Extended Reality for Mental Health Evaluation —A Scoping Review

Olatunji Omisore, PhD^1^; Ifeanyi Odenigbo, BSc^2^; Joseph Orji, BSc^2^; Amelia Beltran, MSc^2^; Meier Sandra, PhD, MD^3^; Nilufar Baghaei, PhD^4^; Rita Orji, PhD^2^

1Shenzhen Institutes of Advanced Technology, Chinese Academy of Sciences, Shenzhen 518055, China.

2Faculty of Computer Science, Dalhousie University, Halifax, Canada

3School of Information Technology and Electrical Engineering, University of Queensland, Brisbane, Australia

4Department of Psychiatry, Dalhousie University, Halifax, Nova Scotia, Canada

**Corresponding Author:**

Olatunji Omisore,

Shenzhen Institutes of Advanced Technology,

Chinese Academy of Sciences, Shenzhen 518055, China.

Phone: 8613172482240

Email: ootsorewilly@gmail.com

| **Checklist item** | **Procedures or guidelines followed** | **Section** |
| --- | --- | --- |
| 1. Title | We identified the need for a scoping review on the development and usage of extended reality for mental health evaluation. The title includes meta information that limits our study to “evaluation” of “extended reality” in “mental health”. | Introduction |
| 2. Abstract | We provided a brief abstract the designs of indicating the inclusion and exclusion rules, the survey method used for analysis, an indication that the differentiate review from existing studies. Also the observations made were reported. |  |
| 3. Objectives | We limit studies to mental health disorder thus gave an oversight on its importance and current status in different countries in the introduction section. Also, a description of our rationale for the review with respect to existing knowledge in mental health assessment was given. This includes explicit statements of study objective(s) or question(s) the review addresses.  The objective is typically to investigate the current adoption and implementation of the different virtual reality, artificial reality, and mixed reality techniques used for evaluating mental health disorder. |  |
| 4. Eligibility criteria | The criteria for inclusion studies to analyze the state-of-the-art in mental health in this review are:   1. articles published between August 2016 and December 2023; and 2. the search terms must be present in the article’ title and/or abstract. | Methods |
| 5. Information sources | The primary sources of information are databases: PubMed, Google Scholar, and ACM Digital Library databases. The three sources were searched several times search a specified period between August 2016 and December 2023. Also, additional 10 articles were identified upon consultation with a field expert on March 3, 2022. |  |
| 6. Search strategy | We used carefully designed search terms defined as “Augmented Reality”, “Mixed Reality”, “Virtual Reality”, “Depression”, and “Anxiety”, “Mental Health” on the three databases. The search terms were chosen as they are most appropriated keywords that reflect the main focus of the review, and have utmost relevance to our research objective. The full terms were exclusively used during the search to avoid any potential conflicts with other terms such as VR intended for “Virtual Reality” extracting use of related acronyms (e.g., Voice Recognition) which would make the filtering cumbersome and not necessarily generate additional useful resources. The selection criteria were carefully designed to consider articles that contain one or multiple search terms in the study’s title and/or abstract sections. Further steps are carried out as explained below |  |
| 7. Selection process | The eligibility criteria were followed to the letter. Thus, we set the exclusion criteria to decide whether a study met at least an inclusion criterion of the review study are removal of:   1. duplicate articles; 2. version updates; 3. articles written in language other than English; 4. studies that reported anxiety or depression as a secondary aspect or induced illness; and 5. articles presenting SWOT analysis, thesis and citations, and scoping reviews.   *The first four authors worked independently and collectively for data creation. These procedures were done with Microsoft Excel and without any form of automation in the process. |  |
| 8. Information collection process and extraction | We carried out data extraction to extract from the final 85 full texts articles obtained after applying the inclusion and exclusion criteria. The articles were downloaded, shared amongst the authors for review and extraction of the following specific details.   1. Publication authors, year, and regions; 2. The study type and study design focus and health domain; 3. Methodology (e.g., study duration, number of sessions, and duration in minutes); 4. The methodology that the study was based on and the evaluation strategy; 5. The VR/AR/MR application and technology (type of headset, toolkit) used for the study; 6. Study demographics such as targeted population, sample size, and age distribution; 7. The motivational strategies, targeted outcome, and region; 8. Key findings on using the XR techniques for managing depression and anxiety.   *The articles processed in a spreadsheet to analyze the adoption of VR, AR, and MR in managing anxiety and depression and related mental health disorders. These procedures were done with Microsoft Excel and without any form of automation in the process. |  |
| 9. Study selection | A flow diagram was designed with the PRISMA guidelines to give visual presentation of the literature screening and selection process. Further studies that apparently met the inclusion criteria were excluded as, upon reading the full texts, they were found irrelevant and/or missing the qualitative data needed. | Methods |
| 10. Study risk of bias assessment and effect measures | Advance search sections of the three databases by four authors individually. The articles located were later combined and processed for duplicates removal. Three of the authors performed the data extraction process while the data validity and accuracy were checked by the fourth author. Tables were created to document the essential characteristics of the 85 studies reported in this review. The table was also used for each synthesis and generation of the statistics (and plots) presented in the manuscript. The main data synthesis typically is replacing missing values with “**Not Specified**”. However, since the age ranges used for defining subjects categories in the 85 articles were not unique, we presented and plotted two age ranges namely:   1. **Children**: 0 — 12 years; **Teenagers**: 10 — 19 years; **Adolescents**: 20 — 25 years; **Adults**: 18 — 65 years; **Older Adults**: 65+ years. This is commonly found in the articles however, there are great overlaps. 2. A second age range was generated by using the range reported in the study, while $\mathrm{mean}_{\mathrm{age}}-SD$— $\mathrm{mean}_{\mathrm{age}}+SD$ was used when only $\mathrm{mean}_{\mathrm{age}}\pm\mathrm{SD}$ was given. SD mean standard deviation.   Subgroup statistical analyses were performed majorly by counting the number of studies that considered the main variables used in this study  These procedures were done with Microsoft Excel and without any form of automation in the process. | Results |
| 11. Results of individual studies | Summary statistics were reported for the information extracted in item 8, while structured tables and plots were used for visual presentation. |  |
| 12. Discussion | We found that the effectiveness of XR technology was focused on anxiety and depression more than other mental health cases thus, we provided a general interpretation of the results in this context with respect to other related studies and evidences that have been reported. | Discussion |
| 13. Recommendation for practice, policy, and future research | . The implications of the results for future studied, development, adoption and usage of extended reality for mental health evaluation in healthcare was given. Also, the limitations of the evidence included and the review processes used in this review were mentioned. | Conclusion and Future Work |
