# Supplementary material for: Extended Reality for Mental Health Evaluation: Scoping Review
Source: JMIR Serious Games. 2024 Jul 24;12:e38413. doi: 10.2196/38413 (PMC11306946; doi:10.2196/38413)
Supplement: Multimedia Appendix 2 [file games_v12i1e38413_app2.docx]

| S/N | Author | | | | | | Title | | | | | | Year | | Type | | | | Design | | | | Method | | | | Aim | | | Findings | | | | | Evaluation Method | | | | | Period | | | | | Session | | | | | Duration | | | | | Audience | | | | | No. of Subjects | | | | | Region | | | | | Age Range | | | | | Health Domain | | | | | | Technology | | | | |
| --- | --- | --- | --- | --- | --- | --- | --- | --- | --- | --- | --- | --- | --- | --- | --- | --- | --- | --- | --- | --- | --- | --- | --- | --- | --- | --- | --- | --- | --- | --- | --- | --- | --- | --- | --- | --- | --- | --- | --- | --- | --- | --- | --- | --- | --- | --- | --- | --- | --- | --- | --- | --- | --- | --- | --- | --- | --- | --- | --- | --- | --- | --- | --- | --- | --- | --- | --- | --- | --- | --- | --- | --- | --- | --- | --- | --- | --- | --- | --- | --- | --- | --- | --- | --- | --- |
| 1 | Appel, Lora et al [26] | | | | | Older Adults With Cognitive and/or Physical Impairments Can Benefit From Immersive Virtual Reality Experiences: A Feasibility Study | | | | 2020 | | | | Analysis | | | | Immersion Analysis | | | | Non Gamified | | | | This paper analysed the feasibility of using VR technology as a tool to provide virtual experience to dependent older adults who have reduced sensory, mobility and/or impaired cognition to promote enjoyments and relaxation, and reduce anxiety and depression. | | | The researchers conducted a study with 66 participants from 4 elderly homes in Ontario. The researchers used a Samsung 360-degree camera to create custom VR films that lasts for 6 minutes but can be repeated within 20 minutes. Each of the participants from the 4 elderly homes experienced the virtual scenes using the Samsung HMD within 8 minutes average. The result shows that over 95% of the participants had no negative side effects when using the VR. Overall, there was a decrease in anxiety and depression, and increase in enjoyment among participants. | | | | | Mixed method | | | | | Not Specified | | | | | 1 | | | | | 20 | | | | | Older Adult | | | | | 66 | | | | | Canada | | | | | 18 to 85 years | | | | | anxiety and depression | | | | | Samsung GearVR HMD | | | | | |  |
| 2 | Martin Dechant, et al [32] | | | | | Potential of virtual reality as a diagnostic tool for social anxiety | | | | 2017 | | | | Experimental | | | | Process Automation | | | | Gamified | | | | To examine differences between low- and high-socially-anxious participants on the basis of specific psychological parameters and recordings of gaze behavior | | | Analyzing fixation durations of faces in a virtual social situation is even more suitable for distinguishing social anxiety disorder | | | | | Qualitative Method | | | | | Not Specified | | | | | Not Specified | | | | | 25 | | | | | Young adults with social anxiety disorder | | | | | 37 | | | | | Germany | | | | | 21.0 ± 2.98 Years | | | | | Social anxiety and social anxiety disorder | | | | | Head-mounted displays | | | | | |  |
| 3 | Bani Mohammad, et al [36] | | | | | Virtual reality as a distraction technique for pain and anxiety among patients with breast cancer: A randomized control trial | | | | 2019 | | | | Experimental | | | | Immersion Analysis | | | | Non Gamified | | | | The purpose of this study is to (1) compare the pain score between patients receiving standard care, which includes pharmacological interventiosn alone, with patients receiving standard care plus VR technology among females who are diagnosed with breast cancer with chronic pain. (2) Compare anxiety level between patients receiving standard care, which includes pharmacological interventions alone, with patients receiving standard care plus VR. | | | Cancer patients suffer from pain and anxiety. Patients who are in pain take painkillers; however, few of the available painkillers achieve adequate pain relief. The immersive VR technology acts as a nonpharmacologic type of analgesia. After running a study with two intervention groups, the following results were obtained. The paired sample test also showed a significant difference in the means of pain scores at the pre and posttest in intervention group and the control group. Regarding the anxiety testing, the independent sample test showed a significant difference postintervention between the two groups. The study suggest that the use of VR with morphine in the intervention roup reduced pain signifiantly more than pharmacological interventions alone in the control group. Immersive VR was an effective distraction technique in this study for anxiety level. | | | | | quantitative Method | | | | | 16 | | | | | 1 | | | | | 30 | | | | | Teenagers and Adult | | | | | 80 | | | | | Jordan | | | | | 18 - 70 years | | | | | Pain and anxiety | | | | | Head-mounted display and headphones (doesn't mention which brand) | | | | | |  |
| 4 | Pinar Bilgin, et al [37] | | | | | A Comparative Study of Mental States in 2D and 3D Virtual Environments Using EEG | | | | 2019 | | | | Experimental | | | | Personalization | | | | Gamified | | | | Aim: to compare the effects of 3D and 2D environment in stress relief and treatments.  Objective: to differentiate the levels of immersiveness and emotion elicitation between 3D-VR and 2D-screen displays using EEG signals | | | Study shows that machine learning method yielded different classification accuracies are obtained for subjects within 2D and 3D environment | | | | | Mixed method | | | | | Not Specified | | | | | Not Specified | | | | | 6 | | | | | Adolescent with Emotion issues | | | | | 10 | | | | | Singapore | | | | | 21-35 years | | | | | Emotion Management | | | | | 2D Monitor; 3D head-mounted VR display | | | | | |  |
| 5 | Elin A Bjarling, et al [38] | | | | | Thought Disposal: Co-Designing a virtual interaction to reduce stress in teens | | | | 2019 | | | | Modeling | | | | Immersion Analysis | | | | Non Gamified | | | | Objective: To develop a VR interaction intended to reduce stress  Hypothesis: Relaxation environment will increase accessibility to mindfulness-based intervention of stress in teens. | | | Participatory approach can aid the design and development of VR system for mindfulness-based intervention in treating mood disorders | | | | | Qualitative Method | | | | | Not Specified | | | | | 3 | | | | | Not Specified | | | | | Children with negative thoughts | | | | | 112 | | | | | USA | | | | | 13 - 19 years | | | | | Negative thoughts | | | | | VR headset | | | | | |  |
| 6 | E Brivio, S Serino, et al [39] | | | | | Virtual reality and 360 panorama technology: a media comparison to study changes in sense of presence, anxiety, and positive emotions | | | | 2021 | | | | Modeling | | | | Immersion Analysis | | | | Non Gamified | | | | The aim of this study is to compare a 360° real panorama environment to a computer-simulated one to verify if they are equally efficient in generating sense of presence, emotions, and relaxation in individuals | | | The study shows that virtual reality exposure therapy and exposure group therapy for social anxiety disorder produce long-lasting benefits, consistent with research on a variety of forms of shortterm cognitive behavioral therapy for social anxiety disorder. | | | | | Mixed method | | | | | Not Specified | | | | | 1 | | | | | 10 | | | | | Young Adults | | | | | 28 | | | | | USA | | | | | 19-69 years | | | | | Social anxiety disorder | | | | | Not Specified | | | | | |  |
| 7 | Chicchi Giglioli IA, et al [40] | | | | | A Novel Integrating Virtual Reality Approach for the Assessment of the Attachment Behavioral System | | | | 2017 | | | | Experimental | | | |  | | | | Gamified | | | | measure real attachment behaviors | | | A significant correlation was found between the participant attachment style and virtual character behaviors across three attachment-related experiences. | | | | | Qualitative Method | | | | | Not Specified | | | | | Not Specified | | | | | Not Specified | | | | | Children | | | | |  | | | | | Spain | | | | | Not mentioned | | | | | Attachment behavior | | | | | Virtual Stealth Attachment | | | | | |  |
| 8 | Dehghan, Fateme et al [41] | | | | | The effect of virtual reality technology on preoperative anxiety in children: a Solomon four-group randomized clinical trial | | | | 2019 | | | | Experimental | | | | Immersion Analysis | | | | Non Gamified | | | | This study investigated the significant effect of using VR technology as an exposure therapy to reduce the anxiety level of children who are about to undergo a surgical operation. | | | The analysis of pre-test and post-test score of one of the intervention group was carried out by the researchers using a Wilcoxon test. It indicated that there is a significant statistical difference in most variables measure. Therefore, their findings shows that there is a significant reduction of fear and anxiety level on preoperative children who were exposed to distraction technique and exposure therapy using VR. The authors further state that their result indicates that the exposure of patients to stress and unfamiliar situations in a safe environment (virtual environment) before the real encounter can help patients to tolerate preoperative anxiety. | | | | | quantitative Method | | | | | Not Specified | | | | | 1 | | | | | 5 | | | | | Children | | | | | 40 | | | | | Iran | | | | | 5 to 9 years | | | | | Anxiety | | | | | VR eyeglass and headphone | | | | | |  |
| 9 | Deppermann S, et al [42] | | | | | Functional co-activation within the prefrontal cortex supports the maintenance of behavioural performance in fear-relevant situations before an iTBS modulated virtual reality challenge in participants with spider phobia | | | | 2016 | | | | Experimental | | | | Immersion Analysis | | | | Non Gamified | | | | 1. The aim of this study was to specifically evaluate the impact of repetitive transcranial magnetic stimulation on emotion regulation in fear-releted situations. | | | 1. The study shows a diminished activation along the left inferior frontal gyrus of participants with spider phobia compared to controls, while a functional connectivity analysis also showed increasedan co-activation between the left inferior frontal gyrus and contra-lateral hemisphere.  2. Behavioural performance was unimpaired in the the phobic and control group, and there was no significant differences in cortical activation between the groups.  3. The prefrontal network gets activated by emotionally-relevant stimuli and supports the maintenance of adequate behavioural reactions. | | | | | Mixed method | | | | | Not Specified | | | | | 1 | | | | | 6 | | | | | Adults | | | | | 83 | | | | | Germany | | | | | 18–65 years | | | | | Spider phobia | | | | | - Head mounted display   - ETG-4000 continuous Optical Topography System | | | | | |  |
| 10 | Donker, Tara et al [43] | | | | | Effectiveness of Self-guided App-Based Virtual Reality Cognitive Behavior Therapy for Acrophobia: A Randomized Clinical Trial | | | | 2019 | | | | Experimental | | | | Personalization | | | | Gamified | | | | This paper tested the effectiveness and user-friendliness of ZeroPhobia, a self-guided VR mobile app installed in users mobile phone and delivered using cardboard VR goggles as a cognitive behaviour therapy (CBT) for reducing acrophobia symptoms in diagnosed patients. | | | The researchers conducted a randomized trial study with 193 participants with acrophobia symptoms and have access to Android smartphone. The participants in intervention group were exposed to the fully guided VR app for 3 weeks. A pretest, posttest, and 3-month follow-up questionnaire were measure for the two groups. The result shows that there was a significant reduction in acrophobia in participants in the intervention group compared to the ones in the control group. The participants in the intervention group rated the VR mobile app as user-friendly and likable. | | | | | quantitative Method | | | | | 3 | | | | | 6 | | | | | 40 | | | | | Adults | | | | | 193 | | | | | Netherland | | | | | 18 to 65 years | | | | | acrophobia symptoms | | | | | Cardboard VR goggle | | | | | |  |
| 11 | Donker, T. Van Esveld, S. Fischer, N. Van Straten, A. [44] | | | | | Phobia - towards a virtual cure for acrophobia: Study protocol for a randomized controlled trial | | | | 2018 | | | | Modeling | | | | Immersion Analysis | | | | Gamified | | | | The aim of this study is to evaluate the effectiveness and user-friendliness of 0Phobia, a gamified self-guided VRET for acrophobia that is delivered through a smartphone app in combination with rudimentary cardboard virtual reality (VR) goggles. | | | There are no findings since they haven't done the evaluation yet | | | | | Mixed method | | | | | 12 | | | | | 2 | | | | | 20 | | | | | Tenagers and Adults | | | | | 180 | | | | | Netherlands | | | | | 18–65 years | | | | | anxiety | | | | | Smarthphone app in combination with rudimentary cardboard virtual reality (VR) googles | | | | | |  |
| S/N | Author | | | | | Title | | | | Year | | | | Type | | | | Design | | | | Method | | | | Aim | | | Findings | | | | | Evaluation Method | | | | | Period | | | | | Session | | | | | Duration | | | | | Audience | | | | | No. of Subjects | | | | | Region | | | | | Age Range | | | | | Health Domain | | | | | Technology | | | | | |  |
| 12 | Hesun Erin et al [45] | | | | | Effectiveness of self-training using the mobile-based virtual reality program in patients with social anxiety disorder | | | | 2017 | | | | Experimental | | | | Immersion Analysis | | | | Non Gamified | | | | To investigate the efficacy of self-training using the newly developed mobile-based virtual reality program for the cost-effective treatment of SAD | | | The SAD patients had greater degree of decrease at the marginal significance level than controls | | | | | quantitative Method | | | | | 2 | | | | | 8 | | | | |  | | | | | Young adults with social anxiety disorder | | | | | 52 | | | | | Korea | | | | | 23 ± 2.6 years | | | | | Social Anxiety Disorder | | | | | Oculus Head-mounted display;  Samsung Gear S2 wrist band;  Earphone | | | | | |  |
| 13 | Yara Fanger, et al [46] | | | | | PIANX â€“ A Platform for Piano Players to Alleviate Music Performance Anxiety Using Mixed Reality | | | | 2020 | | | | Experimental | | | | Immersion Analysis | | | | Non Gamified | | | | to help pianists cope with their musical performance anxiety by using stage fright evoking simulations | | | the study revealed participants’ desired feedback variables to improve their MPA coping skills | | | | | quantitative Method | | | | | Not Specified | | | | | Not Specified | | | | | 20 | | | | | Adult Music Performers | | | | | 23 | | | | | Germany | | | | | 20 - 62 Years | | | | | Anxiety | | | | | HTC Vive PRO VR;  OptiTrack Motion Capture System; | | | | | |  |
| 14 | Gamito, Pedro et al [47] | | | | | Virtual reality cognitive training among individuals with alcohol use disorder undergoing residential treatment: Pilot randomized controlled trial | | | | 2021 | | | | Experimental | | | | Immersion Analysis | | | | Gamified | | | | to explore whether a cognitive training approach using VR exercises based on activities of daily living is feasible for improving the cognitive function of patients with ALcohol use disorder (AUD) undergoing residential treatment, as well as to estimate the effect size for this intervention to power future definitive randomized control trial (RCTs) | | | the data suggest that VR-based cognitive training results in specific contributions to improving attention ability and cognitive flexibility of patients recovering from AUD | | | | | quantitative Method | | | | | 5 | | | | | 10 | | | | | 90 | | | | |  | | | | | 36 | | | | | Portugal | | | | | 18 Years and Above | | | | | Alcoholic Use disorder | | | | | Unity3D Technology | | | | | |  |
| 15 | Alexandra Ghiţă 1, et al [48] | | | | | Attentional Bias, Alcohol Craving, and Anxiety Implications of the Virtual Reality Cue-Exposure Therapy in Severe Alcohol Use Disorder: A Case Report | | | | 2021 | | | | Experimental | | | | Personalization | | | | Gamified | | | | To test the effectiveness of a Virtual Reality Cue-Exposure Therapy (VR-CET) to reduce levels of alcohol craving and anxiety and prompt changes in attentional bias toward alcohol content | | | Pre and post assessment sessions indicated falls on the scores of all instruments assessing alcohol craving, anxiety, and attentional bias. | | | | | quantitative Method | | | | | 5 | | | | | 2 | | | | | 60 | | | | | Adult | | | | | 1 | | | | | Spain | | | | | 49 Years | | | | | Alcohol Use Disorder | | | | | Oculus Touch controllers. | | | | | |  |
| 16 | Jeffrey I Gold, et al [49] | | | | | "Doc McStuffins: Doctor for a Day" Virtual Reality (DocVR) for Pediatric Preoperative Anxiety and Satisfaction: Pediatric Medical Technology Feasibility Study | | | | 2021 | | | | Experimental | | | | Immersion Analysis | | | | Gamified | | | | The aims are to examine the feasibility and efficacy of DocVR for preoperative anxiety, and to improve patient, caregiver, and health care provider satisfaction with the preoperative experience | | | The VR experience resulted in a decrease in overall anxiety and an increase in overall positive affect during the preoperative time. Patients also responded positively to the game, confirming their interest in the content and affirming the quality of the DocVR experience. | | | | | quantitative Method | | | | | Not Specified | | | | | Not Specified | | | | | 55 | | | | | Infants | | | | | 51 | | | | | USA | | | | | 6 - 14 years | | | | | Anxiety | | | | | Headset, handheld controllers | | | | | |  |
| 17 | Hong, Yeon Ju Kim, Hesun Erin Jung, Young Hoon Kyeong, Sunghyon Kim, Jae Jin [50] | | | | | Usefulness of the Mobile Virtual Reality Self-Training for Overcoming a Fear of Heights | | | | 2017 | | | | Experimental | | | | Immersion Analysis | | | | Non Gamified | | | | A mobile virtual reality system, equipped with built-in variables such as heart rate (HR), gaze-down data, and subjective fear rating, can allow individuals with a fear of heights to overcome it by self-training. The VR system presents different missions where users will see different environments in the heights | | | In the present study, the AQ-anxiety scores decreased after self-training in both the low- and high-fear groups, suggesting that our program may be effective in reducing scores on the fear of heights in most people. As expected, the reduction was significantly greater in the high-fear group than in the low-fear group. Both groups showed increases of HR during challenge compared with baseline due to the fear of heights in most missions. Meanwhile, the high-fear group showed significantly greater subjective fear ratings in all missions than the low- fear group. The high-fear group showed significantly less gaze-down percentage than the low-fear group | | | | | quantitative Method | | | | | 2 | | | | | 4 | | | | | 5 | | | | | Adolescents | | | | | 48 | | | | | Korea | | | | | 22 - 25 years | | | | | anxiety | | | | | Mobile phone Glaxy S6 and HMD Gear VR Samsung Electronics, in-ear earphones | | | | | |  |
| 18 | Ji-Won Hur, et al [51] | | | | | Virtual Reality-Based Psychotherapy in Social Anxiety Disorder: fMRI Study Using a Self-Referential Task | | | | 2021 | | | | Experimental | | | | Immersion Analysis | | | | Gamified | | | | The objective of this study waas to determine the changes in self-referential processing and their neural mechanisms following virtual reality treatmen | | | The study shows that participants with social anxiety disorder have increased neural responses during positive self-referential processing in the medial temporal and frontal cortexes compared with those in the control group | | | | | Qualitative Method | | | | | Not Specified | | | | | 6 | | | | | 16 | | | | | Transitional Adult | | | | | 43 | | | | | Korea | | | | | 20 - 27 | | | | | Social anxiety disorder | | | | | VR headse | | | | | |  |
| 19 | M Inozu, et al [52] | | | | | Assessment of virtual reality as an anxiety and disgust provoking tool: The use of VR exposure in individuals with high contamination fear | | | | 2021 | | | | Experimental | | | | Process Automation | | | | Non Gamified | | | | The study examined whether four virtual scenarios that were related to contamination concerns evoked distressing emotions and the urge to wash in individuals with high and low contamination fear. | | | VR tasks successfully induced anxiety, disgust, and the urge to wash in both the HCF and LCF groups | | | | | quantitative Method | | | | | Not Specified | | | | | Not Specified | | | | | 30 | | | | | Young Adults | | | | | 66 | | | | | Turkey | | | | | 18 - 31 Years | | | | | Fear | | | | | head-mounted display, two controllers, and two light emitters for tracking the location and orientation of the head-mounted display and the hand controllers | | | | | |  |
| 20 | Hyu Seok Jeong et al [53] | | | | | Appropriate Number of Treatment Sessions in Virtual Reality-Based Individual Cognitive Behavioral Therapy for Social Anxiety Disorder | | | | 2021 | | | | Experimental | | | | Immersion Analysis | | | | Non Gamified | | | | The purpose of this study was to find out whether VR-based individual CBT with relatively few treatment sessions is effective in improving social anxiety disorder | | | Not Specified | | | | | quantitative Method | | | | | 10 | | | | | 10 | | | | | 30 | | | | | Young Adult | | | | | 115 | | | | | Korea | | | | | 21 - 37 Years | | | | | social anxiety disorder | | | | | automatic monitoring system of the participants' eye movement, speaking time, and heart rate for immediate feedback | | | | | |  |
| 21 | Oswald D Kothgassner et al [54] | | | | | Salivary cortisol and cardiovascular reactivity to a public speaking task in a virtual and real-life environment | | | | 2016 | | | | Experimental | | | | Personalization | | | | Gamified | | | | To examine self-reported, autonomic and endocrine stress responses to a 5-min public speaking task | | | There were no significant differences between the three experimental groups or sexes regarding their experience as a speaker assessed as the number of presentations held over the period of the last 5 years | | | | | Qualitative Method | | | | | Not Specified | | | | | 2 | | | | | 20 | | | | | Younger Adults | | | | | 66 | | | | | Austria | | | | | 20 - 33 years | | | | | Adolescent with public speaking anxiety | | | | | OLED displays  Wireless Gamepad | | | | | |  |
| 22 | Levy F, et al [55] | | | | | E-virtual reality exposure therapy in acrophobia: A pilot study | | | | 2016 | | | | Experimental | | | | Immersion Analysis | | | | Non Gamified | | | | Aim: The aim of the study was to assess e-virtual reality in an acrophobic population.   Hypothesis: The effectiveness of virtual reality exposure therapies were carried out in both traditional and remote sessions. | | | 1. Comparative analysis of the two methods with measures such as anxiety, presence, therapeutic alliance were used to shows that e-Virtual reality can offer similar treatment effects as traditional approach for acrophobic disorders.  traditional sessions in the physical presence of the therapist.  2. No significant difference was found between e-VRET and p-VRET on heart rate, pre-exposure metric scores and before-and-after difference in anxiety VAS ratings | | | | | Mixed method | | | | | 3 | | | | | 6 | | | | | Not Specified | | | | | Adult | | | | | 6 | | | | | France | | | | | 18–65 years | | | | | Acrophobia | | | | | 1. Sensorized Sony head-mounted display  2. directional microphone  3. Webcam | | | | | |  |
| 23 | Stefan Liszio, et al [56] | | | | | A Universe Inside the MRI Scanner: An In-Bore Virtual Reality Game for Children to Reduce Anxiety and Stress | | | | 2020 | | | | Experimental | | | | Immersion Analysis | | | | Gamified | | | | To validate if playing or just watching an animated space story in VR provides distraction and relaxation to the patients. | | | Playful VR application reduced the stress and fear of children related to the MRI exam | | | | | Qualitative Method | | | | | Not Specified | | | | | Not Specified | | | | | 5 | | | | | Children with MRI anxiety | | | | | 15 | | | | | Germany | | | | | 5 - 15 Years | | | | | Anxiety | | | | | Head mounted display | | | | | |  |
| S/N | Author | | | | | Title | | | | Year | | | | Type | | | | Design | | | | Method | | | | Aim | | | Findings | | | | | Evaluation Method | | | | | Period | | | | | Session | | | | | Duration | | | | | Audience | | | | | No. of Subjects | | | | | Region | | | | | Age Range | | | | | Health Domain | | | | | Technology | | | | | |  |
| 24 | Stefan Liszio and Maic Masuch [57] | | | | | Virtual Reality MRI: Playful Reduction of Children's Anxiety in MRI Exams | | | | 2017 | | | | Analysis | | | | Process Automation | | | | Gamified | | | | To present a playful VR application to counter anxiety and avoid sedation of children during MRI examination. | | | Playful VR application reduced the stress and fear of children related to the MRI exam | | | | | Qualitative Method | | | | | Not Specified | | | | | 1 | | | | | 5 | | | | | Children with MRI anxiety | | | | | 13 | | | | | Germany | | | | | 11.0 ± 2.41 Years | | | | | Anxiety | | | | | Head mounted display | | | | | |  |
| 25 | Josephine McInerney et al [58] | | | | | Does raising heart rate prior to a behavioural test enhance learning in cognitive therapy for anxiety? An experimental test for the treatment of fear of heights using virtual reality | | | | 2021 | | | | Experimental | | | |  | | | | Gamified | | | | The aim of the study was to test whether increasing physiological arousal using exercise increases the benefits of behavioural tests using virtual reality. | | | The study shows that An increase in physiological arousal achieved via exercise did not enhance cognitive change in beliefs about feared stimuli. Hence, heart rate was significantly higher in the exercise group throughout compared with the control group | | | | | Qualitative Method | | | | | 6 | | | | | 1 | | | | | 30 | | | | | Young Adults | | | | | 60 | | | | | UK | | | | | 18 - 65 Years | | | | | Acrophobia | | | | | Head-mounted display | | | | | |  |
| 26 | McLay RN, et al [59] | | | | | A Randomized, Head-to-Head Study of Virtual Reality Exposure Therapy for Posttraumatic Stress Disorder | | | | 2017 | | | | Experimental | | | |  | | | | Non Gamified | | | | 1. To determine if virtual reality provide improved outcomes over the conventional control exposure therapy used for Posttraumatic Stress Disorder. | | | 1. The study findings show that VR could improve the identified symptoms when treatments were administered alongside.   2. Virtual Reality Exposure Therapy achieved a 31% improvement on the clinician-administered PTSD cases, while the conventional approach was relatively higher at 37%.  3. This study supported the utility of exposure therapy for PTSD, but did not support additional benefit by the inclusion of virtual reality. | | | | | Mixed method | | | | | 9 | | | | | 12 | | | | | 90 | | | | | Military members with established diagnoses of PTSD | | | | | 153 Consented  81 Participated | | | | | USA | | | | | 18–60 years | | | | | Posttraumatic Stress Disorder | | | | | 3D headmounted display with relevant sounds | | | | | |  |
| 27 | Adrián Montesano et al [60] | | | | | Does Virtual Reality Increase the Efficacy of Psychotherapy for Young Adults With Mild-to-Moderate Depression? A Study Protocol for A Multicenter â€; | | | | 2021 | | | | Experimental | | | | Immersion Analysis | | | | Non Gamified | | | | This project aims to improve the psychological treatment of mild-to-moderate depression in young adults by testing out the efficacy of virtual reality-enhanced personal construct therapy, as compared to personal construct therapy alone and to the reference standard cognitive behavioral therapy | | | this study allowprovidede how to validate using VR-enhanced personal construct therapy as a more efficacious in the treatment of mild-tomoderate depression of young adults than PCT alone and the well-established CBT. | | | | | quantitative Method | | | | | 12 | | | | | 10 | | | | | 60 | | | | | Young Adults | | | | | 225 | | | | | Spain | | | | | 18 - 29 years | | | | | Depression | | | | | Oculus Go or Oculus Quest Head-mount displays. | | | | | |  |
| 28 | Seulki Min, et al [61] | | | | | Effects of Immersive Virtual Reality Content Type to Mindfulness and Physiological | | | | 2020 | | | | Experimental | | | | Immersion Analysis | | | | Non Gamified | | | | To assess how being subjected to imerssion levels modulate anxiety and its psychological and physiological effects on subjects. | | | The VR and study designs can be used for moderating pre-procedural or intraprocedural anxiety | | | | | quantitative Method | | | | | Not Specified | | | | | Not Specified | | | | | 20 | | | | | Adults | | | | | 25 | | | | | Korea | | | | | 19 - 35 years | | | | | For anxiety management in cardiac patients | | | | | Head mounted display; Oculus CV1 | | | | | |  |
| 29 | Neda Keshavarz, et al [62] | | | | | Efficacy of Virtual Reality Based Worry Exposure Therapy on the Anxiety Severity and Worry in Generalized Anxiety Disorder | | | | 2021 | | | | Experimental | | | | Immersion Analysis | | | | Gamified | | | | Thiestudy aimed to evaluate the efficacy of virtual reality-based worry exposure therapy on the Anxiety Severity and worry in patients with symptoms of generalized anxiety disorder | | | virtual reality-based worry exposure therapy has significant efficiency on the reduction of Anxiety Severity and worry clinically and statistically | | | | | quantitative Method | | | | | 2 | | | | | 15 | | | | | 60 | | | | | Young Adult | | | | | 3 | | | | | Iran | | | | | 25 - 36 Years | | | | | Generalized anxiety disorder | | | | | Virtual reality glasses  Samsung Gear VR  headset  (smartphone-based) | | | | | |  |
| 30 | Niharika, Puppala et al [63] | | | | | Effects of distraction using virtual reality technology on pain perception and anxiety levels in children during pulp therapy of primary molars | | | | 2018 | | | | Experimental | | | | Immersion Analysis | | | | Non Gamified | | | | The aim of this study was to evaluate the influence of using virtual reality (VR) eyeglasses on severity of pain and anxiety during pulp therapy in pediatric patients considering childhood anxiety-related disorders as an important confounding factor in the dental setting. | | | The study shows significant decrease in pain perception and state anxiety scores with the use of VR eyeglasses during dental treatment. It is a safe noninvasive technique that does not require any previous education and training and has lasting effects | | | | | Mixed method | | | | | 1 | | | | | 3 | | | | | 45 | | | | | Children | | | | | 40 | | | | | India | | | | | 4 - 8 years | | | | | Pain and anxiety | | | | | Google VR Box, Anti Tank Virtual Reality 3D Glasses, and headphones | | | | | |  |
| 31 | Rahani, Vida Kabiri et al [64] | | | | | Claustrophobia game: Design and development of a new virtual reality game for treatment of claustrophobia | | | | 2018 | | | | Experimental | | | | Personalization | | | | Gamified | | | | The purpose of this research is design and develop a software game called “Claustrophobia Game” for treatment of claustrophobia using VR | | | The obvious anxiery after playing the game was less than before playing the game. It shows that the game can help users to decrease their fear from the closed spaces. | | | | | Qualitative Method | | | | | Not Specified | | | | | Not Specified | | | | | Not Specified | | | | | Teenagers and Adult | | | | | 33 | | | | | Iran | | | | | 15 - 36 Years | | | | | Claustrophobia | | | | | Personal Computer PC platform for running the game software and a head mounted display glasses Oculus Rift | | | | | |  |
| 32 | Shotaro Shimizu, et al [65] | | | | | Influence of Interactive Questions on the Sense of Presence and Anxiety in a Virtual-reality Job-interview Simulation | | | | 2019 | | | | Experimental | | | | Process Automation | | | | Non Gamified | | | | Aim: to help people overcome this anxiety by using a virtual reality job-interview simulation system.  Hypothesis: Existing job-interview simulation systems do not provide sufficiently realistic real-time interactions because only predetermined questions are presented to the users. | | | develop ing questions using the user’s answers is more likely to provoke anxiety and make users feel a sense of presence | | | | | quantitative Method | | | | | Not Specified | | | | | Not Specified | | | | | 5 | | | | | Young Adults on interview | | | | | 22 | | | | | Japan | | | | | 21.5 ± 1.07 years | | | | | Social anxiety disorder | | | | | Electrodermal activity monitor | | | | | |  |
| 33 | Rannveig Sigurvinsdottir, et al [66] | | | | | Social Anxiety, Fear of Negative Evaluation, and Distress in a Virtual Reality Environment | | | | 2021 | | | | Experimental | | | | Personalization | | | | Non Gamified | | | | to examine if giving a presentation in front of a virtual audience induced distress among undergraduate students and to test the hypothesis that FNE would mediate the relationship between social anxiety and distress. | | | Findings indicate that FNE could be a useful treatment target to reduce distress when presenting in front of an audience, either in VR or in person. | | | | | quantitative Method | | | | | Not Specified | | | | | Not Specified | | | | | Not Specified | | | | | Young Adults | | | | | 58 | | | | | USA | | | | | 19 - 35 Years | | | | | Social Anxiety and Distress | | | | | HTC Vive headset. | | | | | |  |
| 34 | Stupar-Rutenfrans, et al [67] | | | | | Beat the Fear of Public Speaking: Mobile 360° Video Virtual Reality Exposure Training in Home Environment Reduces Public Speaking Anxiety | | | | 2017 | | | | Experimental | | | | Immersion Analysis | | | | Non Gamified | | | | The article aims to increase the understanding of how mobile virtual reality exposure therapy VRET can help reduce speaking anxiety | | | Participants with initially high speaking anxiety scored higher on speaking anxiety total score and all speaking anxiety subscales. Participants scored lower on speaking anxiety total score after the PST was completed. For moderate speaking anxiety participants, only the subscale public speaking showed a decrement over time. | | | | | quantitative Method | | | | | 4 | | | | | 3 | | | | | 5 | | | | | Adolescents | | | | | 72 | | | | | Netherlands | | | | | 19 - 25 years | | | | | anxiety | | | | | 360° live recorded VR environments, smartphone VR head-mounted device | | | | | |  |
| 35 | Ramesh Tadayon, et al [68] | | | | | Do Trait Anxiety Scores Reveal Information About Our Response to Anxious Situations? A Psycho-Physiological VR Study | | | | 2019 | | | | Experimental | | | | Immersion Analysis | | | | Gamified | | | | To investigate the potential relationships between anxiety and physiological and perceived reactions to a simulated virtual reality experience that induces mild anxiety;  To investigate the ability to recover from an anxious event in a VRE | | | Study shows the ability to recover from different anxiety levels that are due to physiological reaction of the body. | | | | | quantitative Method | | | | | Not Specified | | | | | Not Specified | | | | | 35 | | | | | Adults with anxiety disorder | | | | | 18 | | | | | USA | | | | | 18 - 60 years | | | | | Not Specified | | | | | HTC Vive, a Wearable Sensing EEG system, a Muse EEG system, an Apple watch, and a Q-sensor | | | | | |  |
| 36 | Tielman, Myrthe L. Neerincx, et al [69] | | | | | A Therapy System for Post-Traumatic Stress Disorder Using a Virtual Agent and Virtual Storytelling to Reconstruct Traumatic Memories | | | | 2017 | | | | Experimental | | | | Immersion Analysis | | | | Non Gamified | | | | The paper presents a system that bridges for patients to follow post-traumatic stress disorder therapy at home. WIth the system, patients can recollec their memories in a digital diary and recreate them in a 3D WorldBuilder. | | | The 3MR_2 system contains two exposure environment, a digital diary and a 3D WorldBuilder in which memories can be recreated. The virtual agent guides and assists patients with their therapy tasks. The system provides safety and reduces human resources. The 3MR_2 provides opportunities to reduce barriers to care; however, is not suitable for all PTSD patients. The participants found usefulness in the system | | | | | Qualitative Method | | | | | Not Specified | | | | | 12 | | | | | Not Specified | | | | | patients with post-traumatic stress disorder | | | | | 4 | | | | | Netherlands | | | | | Not mentioned | | | | | posttraumatic stress disorder | | | | | 3D world builder | | | | | |  |
| S/N | Author | | | | | Title | | | | Year | | | | Type | | | | Design | | | | Method | | | | Aim | | | Findings | | | | | Evaluation Method | | | | | Period | | | | | Session | | | | | Duration | | | | | Audience | | | | | No. of Subjects | | | | | Region | | | | | Age Range | | | | | Health Domain | | | | | Technology | | | | | |  |
| 37 | David Tomasi, et al [70] | | | | | Olfactory Virtual Reality (OVR) for Wellbeing and Reduction of Stress, Anxiety and Pain | | | | 2021 | | | | Discussion | | | | Immersion Analysis | | | | Non Gamified | | | | This study investigates the utilization of OlfactoryVirtual Reality in an inpatient psychiatry unit | | | The research yielded positive outcomes in all areas investi-gated, despite challenges related to the utilization of the device itself,issues in individual olfactory threshold, and COVID-19 restrictions andlimitations. | | | | | quantitative Method | | | | | 4 | | | | | 4 | | | | | 60 | | | | | Young Adults | | | | | 60 | | | | | USA | | | | | 18+ | | | | | Anxiety | | | | | Head-mounteddisplay | | | | | |  |
| 38 | Trahan, Mark H. et al [71] | | | | | Virtual Reality Exposure Simulation for Student Veteran Social Anxiety and PTSD: A Case Study | | | | 2021 | | | | Experimental | | | | Manual | | | | Non Gamified | | | |  | | |  | | | | | Mixed method | | | | | 4 | | | | | 12 | | | | | 25 | | | | | Student veterans | | | | | 1 | | | | | USA | | | | | 36 | | | | | Social Anxiety Disorder | | | | | VR headset and mobile phone | | | | | |  |
| 39 | van 't Wout-Frank, et al [72] | | | | | Combined transcranial direct current stimulation with virtual reality exposure for posttraumatic stress disorder: Feasibility and pilot results | | | | 2019 | | | | Experimental | | | | Immersion Analysis | | | | Non Gamified | | | | Examined the feasility of simultaneous transcranial direct current stimulations (tDCS) application during virtual reality (VR) to reduce psychophysiological arousal and symptoms in Veterans with PTDS. | | | The study demonstrates feasibility of applying tDCS during VR. tDCS + VR has the potential to improve psychophysiological arousal and clinical symptoms of PTSD. | | | | | quantitative Method | | | | | 2 | | | | | 6 | | | | | 25 | | | | | Adolescents and Adults | | | | | 20 | | | | | USA | | | | | 30 - 53 years | | | | | posttraumatic stress disorder | | | | | electrodes, VR environment | | | | | |  |
| 40 | Alexandra Voinescu, et al [73] | | | | | The effectiveness of a virtual reality attention task to predict depression and anxiety in comparison with current clinical measures | | | | 2021 | | | | Experimental | | | | Process Automation | | | | Non Gamified | | | | To clarify whether a whether a VR assessment can predict depression and anxiety with the same or higher level of efectiveness and adherence as classical neuropsychological measures | | | VR is a safe, enjoyable, efective and more ecological alternative for the assessment of attention and inhibition among individuals with elevated anxiety and depression symptoms | | | | | Mixed method | | | | | Not Specified | | | | | 1 | | | | | 5 | | | | | Young Adults | | | | | 82 | | | | | Romania | | | | | 19 - 61 Years | | | | | anxiety and depression | | | | | VR headset | | | | | |  |
| 41 | Joanneke Weerdmeester, et al [74] | | | | | A Randomized Controlled Trial Assessing the Efficacy of a Virtual Reality Biofeedback Video Game: Anxiety Outcomes and Appraisal Processes | | | | 2021 | | | | Experimental | | | | Immersion Analysis | | | | Gamified | | | | The primary aim of this study was to assess the efficacy of a VR biofeedback video game (DEEP) in reducing anxiety symptoms. Our second aim was to assess changes in engagement and cognitive appraisals including self-efficacy, locus of control, and threatchallenge appraisals and explore how these factors relate to anxiety regulation | | | All participants decreased in state anxiety from pre- to postsession | | | | | Mixed method | | | | | 12 | | | | | 2 | | | | | 180 | | | | | Young Adults | | | | | 97 | | | | | Netherlands | | | | | 18 - 23 Years | | | | | Anxiety | | | | | a HTC Vive VR headset and the DEEP controller | | | | | |  |
| 42 | Yukun Xia, Zijie Ding & Yan Gan [75] | | | | | Gamification App Design Based on Augmented Reality Technique for Depression Rehabilitation | | | | 2023 | | | | Modeling | | | | Personalization | | | | Gamified | | | | To design an augmented reality technology with gamified recovery aid for the rehabilitation of depression. | | | Not Listed | | | | | Not Specified | | | | | Not Specified | | | | | Not Specified | | | | | Not Specified | | | | | Not Specified | | | | | Not Specified | | | | | China | | | | | Not Specified | | | | | Depression | | | | | Not Specified | | | | | |  |
| 43 | P. Lindner, J. Dagöö, W. Hamilton, A. Miloff, G.  Andersson, A. Schill, and P. Carlbring [76] | | | | | Virtual Reality exposure therapy for public  speaking anxiety in routine care: a single-subject effectiveness trial | | | | 2021 | | | | Experimental | | | | Immersion Analysis | | | | Non Gamified | | | | To examine the effectiveness of a VR-assisted treatment protocol for public speaking anxiety in routine care. | | | Using a single-subject design and dual-slope modeling (adjusting the treatment-onset slope for treatment effects), we found a significant, large decrease in self-rated public speaking anxiety following the primary three-hour session, similar in magnitude to the previous efficacy trial. | | | | | Mixed Method | | | | | 4 | | | | | 1 | | | | | 180 | | | | | Young Adults | | | | | 23 | | | | | Sweden | | | | | 18+ | | | | | Public  speaking anxiety | | | | | VR Glass  Mobile VR platform, the Oculus Go | | | | | |  |
| 44 | Zhimeng Wang et al [77] | | | | | The Effect of Restorative Environment and Presence Based on Virtual Reality for Anxiety and Depression | | | | 2021 | | | | Experimental | | | | Immersion Analysis | | | | Non Gamified | | | | Explored how to design and develop VR psychological intervention scenarios for people with anxiety and depression mood, and optimized related products or services. | | |  | | | | | quantitative Method | | | | | Not Specified | | | | | Not Specified | | | | | 10 | | | | | Undergrad Students | | | | | 369 | | | | | China | | | | | 17 - 22 Years | | | | | Anxiety | | | | | electroencephalogram (EEG) and electromyography (EMG) | | | | | |  |
| 45 | Brás, Susana Soares, et al [78] | | | | | The feasibility of an augment reality system to study the psychophysiological correlates of fear-related responses | | | | 2018 | | | | Experimental | | | | Immersion Analysis | | | | Non Gamified | | | | Tets the feasibility of an AR system interated in a mobile wearable device for assessing the psychophysiological mechanisms (heart rate) involved in fear responses in real-life contexts. | | | The stimuli presented using AR could indeed induce physiological alteratons in the participants. AR and VR can be optimal solutions for counteracting the effects of in vivo exposure. Both offer high levels of immersion. This system can be apploed to different phobias | | | | | Mixed method | | | | | Not Specified | | | | | Not Specified | | | | | Not Specified | | | | | Tenagers and Adults | | | | | 20 | | | | | Portugal | | | | | 18 - 54 years | | | | | Anxiety | | | | | GPS, accelerometer, gyroscope, camera provided by the smartphone | | | | | |  |
| 46 | Tanae Alicia-Adams Traister [79] | | | | | Virtual Reality Simulation's Influence on Nursing Students' Anxiety and Communication Skills With Anxious Patients: A Pilot Study | | | | 2023 | | | | Experimental | | | | Immersion Analysis | | | | Non Gamified | | | | To determine if full immersion VRS influenced nursing students’ anxiety levels and communication skills when caring for anxious patients | | | 1. The study found that nursing students’ anxiety levels decreased over time while using full-immersion VRS.  2. Comparatively, this study found that full immersion VRS may be an integral tool to reduce students’ anxiety levels and evaluate their performance. | | | | | Quantitative Method | | | | | 4 weeks | | | | | 2 Sessions | | | | | 20 Minutes | | | | | Younger Adults | | | | | 33 | | | | | United States | | | | | 18-46 years | | | | | Anxiety | | | | | VR headset and hand controls | | | | | |  |
| 47 | Dino Krupić, Barbara Zuro, Philip J. Corr [80] | | | | | Anxiety and threat magnification in subjective and physiological responses of fear of heights induced by virtual reality | | | | 2021 | | | | Experimental | | | | Personalization | | | | Non Gamified | | | | The goal is to show how virtual reality can be used for anxiety and threat magnification in subjects with dfear of heights and evlaute the physiological responses. | | | Results showed that subjective levels of distress increased and EDA decreased during induced fear of heights. Furthermore, threat magnification mediated the relationship between anxiety and (a) physiological arousal and (b) subjective distress | | | | | quantitative Method | | | | | Not Specified | | | | | Not Specified | | | | | 3 | | | | | Young Adults | | | | | 122 | | | | | Virtual | | | | | 19 - 45 Years | | | | | Anxiety | | | | | HTC Vive Headset | | | | | |  |
| 48 | Florian Grieger, et al [81] | | | | | Trash It, Punch It, Burn It: Using Virtual Reality to Support Coping with Negative Thoughts | | | | 2021 | | | | Experimental | | | | Personalization | | | | Non Gamified | | | | To investigate if/how VR-baed interventions can support coping with negative thoughts and emotions triggered by everyday textual messages | | | Personalized VRs can aid a general positive shift in thoughts and emotions mainly in the form of increased relaxation and self-refection | | | | | Not Specified | | | | | Not Specified | | | | | 2 | | | | | 5 | | | | | People with negative thoughts | | | | | 10 | | | | | Germany | | | | | 21 - 64 years | | | | | Negative thoughts | | | | | Email | | | | | |  |
| 49 | Karlo Miguel R De Asis, et al [82] | | | | | Serenity: A Stress-Relieving Virtual Reality Application based on Philippine Environmental Variables | | | | 2020 | | | | Experimental | | | | Immersion Analysis | | | | Gamified | | | | To develop a mobile virtual reality that can be used to reduce concurrent stress among class students | | | VR can help promote relaxation to minimize and alter stressful activities and environment | | | | | quantitative Method | | | | | Not Specified | | | | | 1 | | | | | 5 | | | | | College students | | | | | 9 | | | | | Philippines | | | | | 15 - 20 | | | | | Stress | | | | | VR head-mounted display, Google Cardboard, and Samsung Gear VR | | | | | |  |
| 50 | Stefan Liszio, et al [83] | | | | | Pengunaut trainer: a playful VR app to prepare children for MRI examinations: in-depth game design analysis | | | | 2020 | | | | Analysis | | | | Process Automation | | | | Gamified | | | | Aim: to reduce anxiety and stress during MRI examinations.  Objective: help children to familiarize themselves with the medical environment so that they can be examined without fear, rendering sedation unnecessary | | | psychological strategies for patient preparation can be combined with motivational elements from applied game design to enhance young patient’s well-being and cooperativeness before and during MRI examinations | | | | | quantitative Method | | | | | 1 | | | | | 15 | | | | | 8 | | | | | Children with MRI anxiety | | | | | 29 | | | | | Germany | | | | | 5-11 years | | | | | Anxiety | | | | | head-mounted display | | | | | |  |
| 51 | YJ Li and HI Luo [84] | | | | | Depression Prevention by Mutual Empathy Training: Using Virtual Reality as a Tool | | | | 2021 | | | | Modeling | | | |  | | | | Gamified | | | | The proposed study aims to establish cognitive empathy and mutual understanding of depressive disorders in patients and caregivers. | | |  | | | | | quantitative Method | | | | | Not Specified | | | | | Not Specified | | | | | Not Specified | | | | | Not Specified | | | | | Not Specified | | | | | China | | | | | Not Specified | | | | | Depression | | | | | VR headsets: Oculus and HTC Vive | | | | | |  |
| 52 | Preethi Premkumar et al [85] | | | | | The Effectiveness of Self-Guided Virtual-Reality Exposure Therapy for Public-Speaking Anxiety | | | | 2021 | | | | Experimental | | | | Process Automation | | | | Non Gamified | | | | This pilot study aimed to determine whether self-guided virtual-reality exposure therapy (VRET) increases exposure to PSA-specific virtual social threats, and (2) reduces anxiety, arousal, heartrate and PSA over repeated exposure | | | Increased self-exposure to virtual social threat from self-guided VRET relieves anxiety and shows immediate reductions in subjective and physiological arousal during application, but also yields sustained improvement in Public-speaking anxiety PSA. | | | | | Mixed Method | | | | | 1 Month | | | | | 2 Sessions | | | | | 20 Minutes | | | | | Young Adults | | | | | 32 | | | | | United Kingdom | | | | | 18-27 years | | | | | Public speaking Anxiety | | | | | VR Google glass  Smartphone  Biometric wristband | | | | | |  |
| S/N | Author | | | | | Title | | | | Year | | | | Type | | | | Design | | | | Method | | | | Aim | | | Findings | | | | | Evaluation Method | | | | | Period | | | | | Session | | | | | Duration | | | | | Audience | | | | | No. of Subjects | | | | | Region | | | | | Age Range | | | | | Health Domain | | | | | Technology | | | | | |  |
| 53 | Błażej Cieślik, et al [86] | | | | | Immersive virtual reality as support for the mental health of elderly women: a randomized controlled trial | | | | 2023 | | | | Experimental | | | | Immersion Analysis | | | | Non Gamified | | | | This study aimed to evaluate the effectiveness of an immersive virtual reality (IVR) intervention that incorporated Japanese garden aesthetics, relaxation, and elements of Erickson’s psychotherapy in alleviating depression and anxiety symptoms among elderly women | | | Authors confirmed that Immersive VR is more effective than group relaxation for reducing anxiety and depressive symptoms among elderly women participating in support group | | | | | Qualitative Method | | | | | 4 weeks | | | | | 8 Sessions | | | | | 40 Minutes | | | | | Older Adult | | | | | 60 | | | | | Poland | | | | | 60-75 years | | | | | Anxiety and Depression | | | | | VRTierOne device  VR HTC VIVE goggles  HTC VIVE controllers | | | | | |  |
| 54 | Asya S. Berberyan, et al [87] | | | | | Virtual Reality as Anxiety Management Tool | | | | 2023 | | | | Experimental | | | | Immersion Analysis | | | | Non Gamified | | | | To what degree can a virtual scenario consisting of a tropical beach be successfully applied for the reduction of state anxiety and negative mood in healthy individuals | | | State anxiety and negative mood can be decreased using a low-cost virtual reality device. It was suggested that virtual scenarios can improve the emotional well-being and the quality of life of the general population | | | | | Qualitative Method | | | | | 1 Week | | | | | 1 Session | | | | | 30 Minutes | | | | | Adult | | | | | 50 | | | | | Armenia | | | | | 18-45 years | | | | | Anxiety | | | | | Standalone Pico Goblin VR headset | | | | | |  |
| 55 | Gloria Mittmann et al [88] | | | | | LINA – A Social Augmented Reality Game Around Mental Health, Supporting Real-World Connection and Sense of Belonging for Early Adolescents | | | | 2022 | | | | Modeling | | | | Process Automation | | | | Gamified | | | | The aim was to design and co-develop a mobile AR serious game (LINA) to address real-world social connection and SB in the classroom with and for early adolescents (aged 10+). | | | Both quantitative and qualitative data from the evaluation study showed high acceptability of the game, and qualitative data show promising results regarding efficacy | | | | | Quantitative Method | | | | | Once | | | | | 1 Session | | | | | 75 Minutes | | | | | Children | | | | | 91 | | | | | Austria | | | | | 10 - 13 years | | | | | Attachment behavior | | | | | Smartphone  Unity Engine | | | | | |  |
| 56 | Bellinger D et al [89] | | | | | The application of virtual reality exposure versus relaxation training in music performance anxiety: a randomized controlled study | | | | 2023 | | | | Experimental | | | | Immersion Analysis | | | | Non Gamified | | | | The study aims to evaluate the effectiveness of VRET for the treatment of Music Performance Anxiety compared to a relaxation technique on anxiety symptoms and corresponding cardiovascular parameters in musicians | | | Regarding music performance anxiety itself, it remains unclear if a music performance on a “virtual stage” in an artificial environment is able to generate the same stress or anxiety level in musicians as in vivo. | | | | | Mixed Method | | | | | Not Specified | | | | | 4 Sessions | | | | | 60 Minutes | | | | | Adult | | | | | 46 | | | | | Germany | | | | | 18-60 years | | | | | Anxiety | | | | | HTC Vive head-mounted display | | | | | |  |
| 57 | Turoń-Skrzypińska et al [90] | | | | | Impact of virtual reality exercises on anxiety and depression in hemodialysis | | | | 2023 | | | | Experimental | | | | Immersion Analysis | | | | Non Gamified | | | | The aim of the study was to assess the relationship between regular physical activity performed with the use of virtual reality and the occurrence of symptoms of anxiety and depression in hemodialysis patients | | | The research showed that regular exercises using virtual reality may be associated with a reduction in the occurrence of anxiety and depression symptoms in patients included in the chronic hemodialysis program | | | | | Qualitative Method | | | | | Not Specified | | | | | Not Specified | | | | | 20 Minutes | | | | | Not Specified | | | | | 85 | | | | | Poland | | | | | 18+ years | | | | | Anxiety and Depression | | | | | Not Specified | | | | | |  |
| 58 | Fuad A. El-Qirem et al [91] | | | | | Effect of virtual reality therapy on stress and anxiety symptoms, and physiological measures among University students: an experimental study in Jordan | | | | 2023 | | | | Experimental | | | | Process Automation | | | | Non Gamified | | | | To assess the effect of virtual reality therapy on the stress and anxiety symptoms. | | | VR lowered the perceived stress and anxiety with statistical significance. The researchers think that using VRT enhanced students psychologically and physiologically in a safe and risk-free therapeutic experience | | | | | Qualitative Method | | | | | 4 weeks | | | | | 2 Sessions | | | | | 15 Minutes | | | | | Young Adults | | | | | 90 | | | | | Jordan | | | | | 18-32 years | | | | | Stress and Amxiety | | | | | HTC Vive head mounted display.  Digital blood pressure machine | | | | | |  |
| 59 | Min-Kyeong Kim, et al [92] | | | | | Neural effects of a short-term virtual reality self-training program to reduce social anxiety | | | | 2020 | | | | Experimental | | | | Process Automation | | | | Non Gamified | | | | This study aimed to neurobiologically verify the possibility of Virtual reality self-training (VRS) as a tool to improve symptoms using functional magnetic resonance imaging (fMRI) with the distress and speech evaluation tasks in patients with social anxiety disorder (SAD) with and without VRS. | | | The post-VRS assessment showed significantly decreased anxiety and avoidance scores, distress index, and negative evaluation index for ‘self’ | | | | | Not Specified | | | | | 2 Weeks | | | | | 8 Sessions | | | | | 21 | | | | | Young Adults | | | | | 61 | | | | | Korea | | | | | 19 - 30 years | | | | | Social Anxiety Disorder | | | | | Occulus VR SmartPhone | | | | | |  |
| 60 | Qian Li and Huajie Sui [93] | | | | | Application of the perspective based on virtual reality technology to relieve anxiety in practice | | | | 2022 | | | | Not Specified | | | | Not Specified | | | | Not Specified | | | | The goal of this study was to implement a highly immersive and non-realistic virtual environment using Seq2seq model to treat patients with anxiety crisis by exposing them to virtual scenes and related stimuli needed to relieve anxiety. | | | After the relevant data statistics, it was found that the participants who received VRET had less anxiety and avoidance behaviors in the treatment of fear of altitude. | | | | | Mixed method | | | | | Not Specified | | | | | Not Specified | | | | | 60 minutes | | | | | Patients with anxiety management crisis. | | | | | 10 | | | | | China | | | | | Not Specified | | | | | Stress Managemnet | | | | | Not Specified | | | | | |  |
| 61 | Sylvie Bernaerts, et al [94] | | | | | Virtual Reality for Distraction and Relaxation in a Pediatric Hospital Setting: An Interventional Study With a Mixed-Methods Design | | | | 2022 | | | | Experimental | | | | Immersion Analysis | | | | Non Gamified | | | | The aim of this study was to investigate the feasibility, acceptability, tolerability (primary outcomes), and preliminary effectiveness (secondary outcome) of Relaxation-VR both from a patient and clinician perspective | | | Results indicate that VR use (in particular, the Relaxation-VR prototype) for both distraction and relaxation is acceptable, feasible and tolerable for a variety of pediatric patients aged 4–16 years, as assessed in both patients and clinicians, and can reduce anxiety, pain and tension (stress), and increase happiness in a hospital setting. | | | | | Mixed method | | | | | Not Specified | | | | | 3 Sessions | | | | | 14 minutes | | | | | Children | | | | | 51 | | | | | Belgium | | | | | 4 - 16 years | | | | | Anxiety | | | | | Oculus Go VR headset | | | | | |  |
| 62 | Cosmin Octavian Popa, et al [95] | | | | | Standard CBT versus integrative and multimodal CBT assisted by virtual-reality for generalized anxiety disorder | | | | 2022 | | | | Analysis | | | | Manual | | | | Non Gamified | | | | The objective of this study was to compare the efficiency of a standard CBT protocol targeting worries, dysfunctional beliefs, and intolerance of uncertainty with an integrative and multimodal CBT intervention with VR | | | Result showed that the CBT interventions for treating adults with moderate Generalized Anxiety Disorder (GAD) symptomatology was effective in reducing the intensity of anxious manifestations and dysfunctional cognitive processes involved in this disorder. | | | | | Quantitative Method | | | | | 10 Weeks | | | | | 10 Sessions | | | | | 60 minutes | | | | | Young Adults | | | | | 66 | | | | | Romania | | | | | 22-25 years | | | | | Generalized anxiety disorder | | | | | Not Specified | | | | | |  |
| 63 | Desirée Colombo , et al [96] | | | | | Behavioral Activation through Virtual Reality for Depression: A Single Case Experimental Design with Multiple Baselines | | | | 2022 | | | | Experimental | | | | Manual | | | | Non Gamified | | | | The aim of this study was to test the effectiveness of a four-session, VR-assisted BA protocol for individuals with moderate-to-severe depressive symptoms. | | | Most participants reported increased daily rates of savoring. In other words, the VR intervention was not only associ-ated with an adaptive behavioral change, but also with a significant improvement in the quality and enjoyment of the daily activities in more than half participants. | | | | | Quantitative Method | | | | | 4 weeks | | | | | 4 Sessions | | | | | 30 - 45 minutes | | | | | Young Adults | | | | | 8 | | | | | Spain | | | | | 20 - 26 years | | | | | Depression | | | | | Google Earth VR for Oculus Rift | | | | | |  |
| 64 | José A. Camacho-Conde, et al [97] | | | | | Assessment of Attentional Processes in Patients with Anxiety-Depressive Disorders Using Virtual Reality | | | | 2021 | | | | Analysis | | | | Manual | | | | Non Gamified | | | | The objective of this study was to characterize the attention deficits in a sample comprising two types of clinical profiles (i.e., affective and anxiety disorder) using a test of continuous VR execution. | | | Results suggest attentional deficits in both clinical populations when performing a continuous performance test that involved the participation of the central executive system of working memory. | | | | | Mixed method | | | | | Not Specified | | | | | Not Specified | | | | | 18 - 24 minutes | | | | | Adults | | | | | 115 | | | | | Spain | | | | | 16 - 70 years | | | | | Anxiety and Depression | | | | | Not Specified | | | | | |  |
| 65 | Johan Lundin, et al [98] | | | | | Using 360-degree videos for virtual reality exposure in CBT for panic disorder with agoraphobia: a feasibility study | | | | 2021 | | | | Modeling | | | | Immersion Analysis | | | | Non Gamified | | | | The aim of this study was to investigate whether VR-CBT using filmed virtual environments produced with a low-cost 360-degree film camera can be a feasible and acceptable treatment for panic disorder with agoraphobia (PDA) when implemented in a primary care context. | | | The results showed that treatment satisfaction was high and participants were significantly improved on PDA-related measures at post-treatment and at 6-month follow-up with large effect sizes. | | | | | Mixed method | | | | | 10 - 12 Weeks | | | | | 12 Sessions | | | | | 60 minutes | | | | | Adults | | | | | 12 | | | | | Sweden | | | | | 23 - 62 years | | | | | Phobia | | | | | HTC Vive head-mounted display  Samsung VR 360 headset | | | | | |  |
| 66 | Janice Tan, et al [99] | | | | | Examining the potential of VR program Tilt Brush in reducing anxiety | | | | 2022 | | | | Experimental | | | | Process Automation | | | | Non Gamified | | | | This study aimed to explore the potential of using VR for therapeutic benefits through examining the level of flow and anxiety-reducing effects of freeform drawing in real life (on paper) versus drawing in VR (using Tilt Brush). | | | Overall level of flow was not significantly different between both groups, implying drawing in VR induces as much flow as drawing in real life. | | | | | Quantitative Method | | | | | Not Specified | | | | | 1 Session | | | | | 60 minutes | | | | | Adults | | | | | 40 | | | | | Australia | | | | | 19 - 68 years | | | | | Anxiety | | | | | Oculus Quest headset. | | | | | |  |
| S/N | Author | | | | | Title | | | | Year | | | | Type | | | | Design | | | | Method | | | | Aim | | Findings | | | | | Evaluation Method | | | | | Period | | | | | Session | | | | Duration | | | | | | Audience | | | | | No. of Subjects | | | | | Region | | | | | Age Range | | | | | Health Domain | | | | | Technology | | | | | |  |  |
| 67 | Marta Modrego-Alarc´on et al [100] | | | | | Efficacy of a mindfulness-based programme with and without virtual reality support to reduce stress in university students: A randomized controlled trial | | | | 2021 | | | | Experimental | | | | Immersion Analysis | | | | Gamified | | | | To evaluate the efficacy of a mindfulness-based programme (MBP) for reducing stress in university students and its action mechanisms and to explore the capacity of virtual reality (VR) exposure to enhance adherence to the intervention. | | | This RCT supports the efficacy of an MBP compared to relaxation for reducing stress in university students through mindfulness and self-compassion as mechanisms of change. VR exposure may enhance treatment adherence. | | | | | Mixed method | | | | | 6 Weeks | | | | | 6 Sessions | | | | | 90 minutes | | | | | Adults | | | | | 280 | | | | | Spain | | | | | 18+ | | | | | Stress Management | | | | | Samsung GEAR VR goggles | | | | | |  |
| 68 | Benjamin Arnfred et al [101] | | | | | Group cognitive behavioural therapy with virtual reality exposure versus group cognitive behavioural therapy with in vivo exposure for social anxiety disorder and agoraphobia: a protocol for a randomised clinical trial | | | | 2021 | | | | Experimental | | | | Process Automation | | | | Non Gamified | | | | The SoREAL-trial aims to investigate the effect of group cognitive behavioural therapy (CBT-in vivo) versus group CBT with virtual reality exposure (CBT-in virtuo) for patients diagnosed with social anxiety disorder and/or agoraphobia, in mixed groups. | | | Total scores on the LSAS for patients with social anxiety disorder and the MIA for patients with agoraphobia measured pretreatment, post-treatment and at 1-year follow-up converted to the POMP and averaged within treatment arms. POMP calculations can bring differently measured items to the same metric and do not change the multivariate distribution and covariance matrix of the transformed variables. Therefore, scales transformed with the POMP method can be used to examine mean-level differences between groups.59 | | | | | Quantitative Method | | | | | 14 weeks | | | | | 14 Sessions | | | | | 120 minutes | | | | | Adult | | | | | 301 | | | | | Denmark | | | | | 18-75 years | | | | | Social anxiety disorder  Agoraphobia | | | | | Not Specified | | | | | |  |
| 69 | Florian P. Binder et al [102] | | | | | Facing Your Fear in Immersive Virtual Reality: Avoidance Behavior in Specific Phobia | | | | 2022 | | | | Modeling | | | | Process Automation | | | | Non Gamified | | | | The goal of this study was to examine the sensitivity and feasibility of automated experimental procedure using immersive virtual reality to assess avoidance behavior in patients with specific phobia. | | | the behavioral tasks are well suited to assess avoidance behavior in phobic participants and provide detailed insights into the process of avoidance. | | | | | Qualitative Method | | | | | Not Specified | | | | | Not Specified | | | | | Not Specified | | | | | Young Adults | | | | | 31 | | | | | Germany | | | | | 18 - 35 years | | | | | Phobia | | | | | Unity 3D Pro HTC Vive Pro  Eye VR goggles  Perception Neuron motion tracking system  ECG Device | | | | | |  |
| 70 | Anne Sophie Hildebrand et al [103] | | | | | Self‑guided virtual reality therapy for social anxiety disorder: a study protocol for a randomized controlled trial | | | | 2022 | | | | Experimental | | | | Immersion Analysis | | | | Non Gamified | | | | The study aims to investigate if ultra-short-time therapy involving self-guided digital therapeutic applications with virtual reality (VR) components can reduce the severity of SAD. | | | 1. Because of only a few appointments with therapists, more patients could be treated by these therapists in equivalent periods. This could reduce waiting times and the risk of pre-therapy attrition and chronic progressions of SAD.   2., patients can complete VR therapy independently of a psychotherapist | | | | | Not Specified | | | | | 12 Weeks | | | | | 4 Sessions | | | | | 200 Minutes | | | | | Not Specified | | | | | 40 | | | | | Germany | | | | | 18+ years | | | | | Social anxiety disorder | | | | | Not Specified | | | | | |  |
| 71 | Kaitlyn Harrison, et al [104] | | | | | The Effectiveness of Virtual Reality on Anxiety and Performance in Female Soccer Players | | | | 2021 | | | | Experimental | | | | Process Automation | | | | Gamified | | | | The purpose of the current study was to examine how a VR relaxation intervention affected perceived anxiety levels and penalty kick performance of female soccer players. | | | The results indicated that the VR intervention significantly reduced the participants’ perceived cognitive and somatic anxiety levels, increased self-confidence, and reduced their HR. However, future research is still needed to understand the relationship between VR relaxation, kinematics, and performance | | | | | Qualitative Method | | | | | Not Specified | | | | | Not Specified | | | | | 10 minutes | | | | | Young Adults | | | | | 13 | | | | | USA | | | | | 19 - 22 years | | | | | Anxiety | | | | | Oculus Quest  Polar H10 sensor  ECG Holter monitor | | | | | |  |
| 72 | Marieke B. J. Toffolo et al [105] | | | | | Automated app-based augmented reality cognitive behavioral therapy for spider phobia: Study protocol for a randomized controlled trial | | | | 2022 | | | | Experimental | | | | Immersion Analysis | | | | Non Gamified | | | | This study will use a Randomized Controlled Trial design to investigate whether ZeroPhobia: Arachnophobia, a 6-week AR Exposure Therapy smartphone self-help application, can effectively reduce spider phobia symptoms. Also, user-friendliness of the app and its effects on treatment outcome were examined | | | Incomplete: "Data collection started September 2021 and we expect to finish in September 2022." | | | | | Mixed Method | | | | | 6 Weeks | | | | | 6 Sessions | | | | | 30 -minutes | | | | | Adult | | | | | 112 | | | | | Netherlands | | | | | 18–64 | | | | | spider phobia | | | | | Augmented Reality | | | | | |  |
| 73 | Mikael Rubin, et al [106] | | | | | Attention guidance augmentation of virtual reality exposure therapy for SAD | | | | 2022 | | | | Experimental | | | | Immersion Analysis | | | | Non Gamified | | | | To investigate attentional avoidance as a potential mechanism for Social anxiety disorder SAD | | | There was strong evidence that our intervention engaged the target mechanism the exposure augmentation led to a meaningful change in attention allocation, with a substantially greater proportion of gaze toward uninterested audience compared to standard exposure group after intervention. | | | | | Qualitative Method | | | | | 2 Weeks | | | | | 2 sessions | | | | | 45 Minutes | | | | | Adult | | | | | 21 | | | | | USA | | | | | 18–65 | | | | | Social Anxiety Disorder | | | | | Samsung Gear 360 camera Oculus Rift VR Headset | | | | | |  |
| 74 | Bokyoung Shin1, et al [107] | | | | | Effectiveness of Self-Guided Virtual Reality–Based Cognitive Behavioral Therapy for Panic Disorder: Randomized Controlled Trial | | | | 2021 | | | | Experimental | | | | Immersion Analysis | | | | Non Gamified | | | | The main goal of this study was to offer data about the efficacy of a mobile app-based self-led VR CBT in the treatment of panic disorder. | | | In within-group analyses, the VR treatment group exhibited improvements in panic disorder symptoms, anxiety, and depression after 4 weeks, while the waitlist group did not show any significant improvement. Compared to the waitlist group, the VR treatment group showed significantly greater improvements in the Panic Disorder Severity Scale in both completer analysis and intention-to-treat analysis. The self-guided, mobile app-based VR intervention was effective in the treatment of panic symptoms and restoring the autonomic nervous system demonstrating the validity of the use of VR for self-guided treatment. VR treatment can be a cost-effective therapeutic approach. | | | | | Mixed Method | | | | | 4 weeks | | | | | 12 sessions | | | | | 30 Minutes | | | | | Adult | | | | | 61 | | | | | Korea | | | | | 19-60 years | | | | | Panic Disorder | | | | | Galaxy 8+ Smartphone  Samsung Gear VR  Insta360 Pro Camera | | | | | |  |
| 75 | Heewon Na, et al [108] | | | | | Mixed Reality-Based Interaction between Human and Virtual Cat for Mental Stress Management | | | | 2022 | | | | Experimental | | | | Immersion Analysis | | | | Non Gamified | | | | This study aimed to analyze the effect of stress reduction using an MR-based HAI. | | | The findings of the study demonstrate that the MR-based interaction with virtual animals significantly reduces mental stress and induces positive emotions. We expect that this study could provide a basis for the widespread use of MR-based content in the field of mental health | | | | | Mixed Method | | | | | Not Specified | | | | | 2 Sessions | | | | | 35 Minutes | | | | | Young Adults | | | | | 30 | | | | | Korea | | | | | 20-25 years | | | | | Stress Reduction | | | | | Microsoft HoloLens  Wireless ECG sensor | | | | | |  |
| 76 | Estrella-Juarez F, et al [109] | | | | | Effect of Virtual Reality and Music Therapy on the Physiologic Parameters of Pregnant Women and Fetuses and on Anxiety Levels: A Randomized Controlled Trial | | | | 2023 | | | | Experimental | | | | Manual | | | | Non Gamified | | | | This study aimed to evaluate the effects of virtual reality and music therapy on anxiety levels, maternal and fetal physiologic parameters, and labor and birth outcomes. | | | This study support the use of music and virtual reality during nonstress tests and labor as nonpharmacologic interventions to reduce anxiety, improve maternal and fetal physiologic parameters, and improve labor and birth outcomes | | | | | Qualitative Method | | | | | Not Specified | | | | | 2 Sessions | | | | | 20 Minutes | | | | | Adult  Pregnant Women | | | | | 343 | | | | | Spain | | | | | 26-36 years | | | | | Anxiety | | | | | Bnext 3D glasses | | | | | |  |
| 77 | Donker T, et al [110] | | | | | Automated mobile virtual reality cognitive behavior therapy for aviophobia in a natural setting: a randomized controlled trial | | | | 2023 | | | | Experimental | | | | Immersion Analysis | | | | Gamified | | | | To assess the effectiveness of a fully automated aviophobia smartphone app treatment | | | 1. VR-CBT app treatment condition exhibited a significant reduction in fear-of-flying symptoms.  2. Baseline aviophobia symptoms influenced treatment effectiveness: participants with more severe fear-of-flying symptoms at baseline benefitted more from the VR-CBT | | | | | Qualitative Method | | | | | 6 Weeks | | | | | 6 Sessions | | | | | 40 Minutes | | | | |  | | | | | 153 | | | | | Germany | | | | |  | | | | | Phobia | | | | | Unity game engine | | | | | |  |
| 78 | Shu Wei, et al [111] | | | | | A randomised controlled test of emotional attributes of a virtual coach within a virtual reality (VR) mental health treatment | | | | 2023 | | | | Experimental | | | | Immersion Analysis | | | | Non Gamified | | | | To test the impact of VR coach positive non-verbal behaviours on the treatment credibility and expectancy in acrophobia treatment. | | | 1. Simple main effects analysis showed that affirmative nods led to significant increases in treatment credibility but that warm facial expressions did not.  2. There was no statistically significant interaction between warm facial expressions and affirmative nods. | | | | | Qualitative Method | | | | | 1 Week | | | | | 1 Session | | | | | 45 Minutes | | | | | Adult | | | | | 120 | | | | | United Kingdom | | | | | 18-70 years | | | | | Acrophobia | | | | | Meta Quest 2 | | | | | |  |
| 79 | Eran Orr, et al [112] | | | | | Virtual reality in the management of stress and anxiety disorders: A retrospective analysis of 61 people treated in the metaverse | | | | 2023 | | | | Experimental | | | | Immersion Analysis | | | | Non Gamified | | | | To determine if virtual mental health treatment was achievable and safe, with measurable outcomes repeated at multiple time points | | | The results of this retrospective analysis of health records strongly suggest that people seeking care for stress and anxiety disorders are willing to consent for treatment and participate for a prescribed course of treatment in the metaverse | | | | | Mixed Method | | | | | 10-25 Weeks | | | | | 4 Sessions | | | | | Not Specified | | | | | Adults | | | | | 61 | | | | | United States, Israel, Australia | | | | | 30-60 years | | | | | Stress and Anxiety | | | | | Pico Neo 2 head mount display; Pico Neo 2 hand controllers | | | | | |  |
| S/N | | Author | | Title | | | | Year | | | Type | | | | | Design | | | | Method | | | | Aim | | | | | | | Findings | | | | | Evaluation Method | | | | | Period | | | | | Session | | | | | Duration | | | | | Audience | | | | | No. of Subjects | | | | | Region | | | | | Age Range | | | | | Health Domain | | | | Technology | | |  |  |  |
| 80 | | | Fatime Zeka, et al [113] | | Examination of gaze behaviour in social anxiety disorder using a virtual reality eye-tracking paradigm: protocol for a case–control study | | | | 2023 | | | Analysis | | | | | Manual | | | | Non Gamified | | | | The study aims to examine gaze behaviour differences in a clinical sample of SAD compared with an HCG using a VR eye-tracking paradigm | | | | | | |  | | | | | Qualitative Method | | | | | 1 Week | | | | | | 3 Sessions | | | | 8 Minutes | | | | | Adults | | | | | 58 | | | | | Denmark | | | | | 18-75 Years | | | | | Social anxiety disorder | | | | | VR head mounted display | | | |
| 81 | | | Jessica Bond et al [114] | | A safe place to learn: a peer research qualitative investigation of automated virtual reality cognitive therapy | | | | 2023 | | | Analysis | | | | | Immersion Analysis | | | | Non Gamified | | | | To use a peer research approach to explore participants’ experiences with gameChange VR therapy | | | | | | | Participants reported the substantial impact of anxious avoidance on their lives before the VR intervention, leaving some of them housebound and isolated. Those who were struggling the most with agoraphobic avoidance expressed the most appreciation for, and gains from, the gameChange therapy | | | | | Qualitative Method | | | | | Not Specified | | | | | | 3 Sessions | | | | 80 Minutes | | | | | Adults | | | | | 20 | | | | | United Kingdom | | | | | 25-60 years | | | | | Phobia | | | | | Not Specified | | | |
| 82 | | | Cameron Lacey et al [115] | | oVRcome – Self-guided virtual reality for specific phobias: A randomised controlled trial | | | | 2022 | | | Experimental | | | | | Immersion Analysis | | | | Non Gamified | | | | To evaluate the effectiveness of a mobile health application combining self-guided virtual reality exposure and cognitive behaviour therapy, for five specific phobias. | | | | | | | The mean change in Severity Measures for Specific Phobia – Adults score from baseline to week 6 was greater in the active group compared with the waitlist group | | | | | Qualitative Method | | | | | 6 weeks | | | | | | 2 Sessions | | | | 10 Minutes | | | | | Adults | | | | | 12 | | | | | New Zealand | | | | | 18-64 | | | | | Phobia | | | | | VR Headset  Smartphone | | | |
| 83 | | | Amanda N Levy et al [116] | | Patient Perceptions of In Vivo Versus Virtual Reality Exposures for the Treatment of Anxiety Disorders: Cross-Sectional Survey Study | | | | 2023 | | | Analysis | | | | | Not Specified | | | | Non Gamified | | | | This study aims to explore the perceptions of individuals with anxiety disorders toward in vivo and VRET. | | | | | | | Our findings can inform therapists about the degree of patient interest in both methods while exploring the demand for VRET as an alternative and novel treatment approach. | | | | | Qualitative Method | | | | | Not Specified | | | | | | Not Specified | | | | Not Specified | | | | | Adults | | | | | 184 | | | | | United States | | | | | 20-40 years | | | | | Anxiety | | | | | Not Specified | | | |
| 84 | | | Jia-Yan Pan [117] | | Internet-Based Cognitive Behavioral Therapy and Virtual Reality Exposure Therapy for Social Anxiety Disorder: Protocol for a Randomized Controlled Trial in Hong Kong | | | | 2023 | | | Experimental | | | | | Immersion Analysis | | | | Gamified | | | | This study aims to develop an iCBT program that includes VRET for Hong Kong adults with SAD. | | | | | | | iCBT serves as a more preferred and accessible treatment option for Hong Kong clients with SAD | | | | | Qualitative Method | | | | | 14 weeks | | | | | | 5 Sessions | | | | 15 Minutes | | | | | Adults | | | | | 166 | | | | | Hong Kong | | | | | 18-70 years | | | | | Social anxiety disorder | | | | | VR Head mount  VR game engine   VR motion tracking | | | |
| 85 | | | Karin Cinalioglu, Harmehr Sekhon, Soham Rej [118] | | Effects of a Virtual Reality Assisted Mindfulness Intervention in Older Adults | | | | 2023 | | | Experimental | | | | | Immersion Analysis | | | | Non Gamified | | | | This study aims to evaluate the feasibility, acceptability, and effects of virtual-reality based mindfulness meditation on stress and depression in older adults | | | | | | | Qualitative analyses through semi-structured interviews and participant observation of participants’ experiences showed that overall satisfaction and perceived benefits were high | | | | | Qualitative Method | | | | | 4 Weeks | | | | | | 8 Sessions | | | | 15 Minutes | | | | | Older Adult | | | | | 30 | | | | | Israel | | | | | 60+ years | | | | | Stress and Depression | | | | | Oculus Quest 2 headset | | | |
